# Epigenetic enzymes influenced by oxidative stress and hypoxia mimetic in osteoblasts are differentially expressed in patients with osteoporosis and osteoarthritis

**DOI:** 10.1038/s41598-018-34255-4

**Published:** 2018-11-01

**Authors:** Peter Vrtačnik, Janja Zupan, Vid Mlakar, Tilen Kranjc, Janja Marc, Barbara Kern, Barbara Ostanek

**Affiliations:** Department of Clinical Biochemistry, Faculty of Pharmacy, University of Ljubljana, Aškerčeva cesta 7, SI-1000 Ljubljana, Slovenia

## Abstract

Epigenetic mechanisms including posttranslational histone modifications and DNA methylation are emerging as important determinants of bone homeostasis. With our case-control study we aimed to identify which chromatin-modifying enzymes could be involved in the pathology of postmenopausal osteoporosis and osteoarthritis while co-regulated by estrogens, oxidative stress and hypoxia. Gene expression of *HAT1*, *KAT5*, *HDAC6*, *MBD1* and *DNMT3A* affected by oxidative stress and hypoxia in an *in vitro* qPCR screening step performed on an osteoblast cell line was analysed in trabecular bone tissue samples from 96 patients. Their expression was significantly reduced in patients with postmenopausal osteoporosis and osteoarthritis as compared to autopsy controls and significantly correlated with bone mineral density and several bone histomorphometry-derived parameters of bone quality and quantity as well as indicators of oxidative stress, RANK/RANKL/OPG system and angiogenesis. Furthermore, oxidative stress increased DNA methylation levels at the *RANKL* and *OPG* promoters while decreasing histone acetylation levels at these two genes. Our study is the first to show that higher expression of *HAT1*, *HDAC6* and *MBD1* is associated with superior quantity as well as quality of the bone tissue having a more favourable trabecular structure.

## Introduction

Epigenetic mechanisms represent an important ubiquitous group of gene expression regulators associated with normal and aberrant bone remodelling and homeostasis^1^. Posttranslational histone modifications and DNA methylation are two of the most studied epigenetic mechanisms. Acetylation of histones is associated with transcriptional activation of target genes and is mediated by histone acetylases (HATs) and histone deacetylases (HDACs). DNA methylation is on the other hand usually associated with target gene repression. It is mediated by DNA methyltransferases (DNMTs) and facilitates binding of various DNA methyl-binding proteins to the DNA molecule^2^. However, any of these epigenetic mechanisms could be modified by different environmental factors, like oxidative stress, hypoxia and estrogens which are some of the known factors influencing bone remodelling in complex *in vivo* conditions.

17β-estradiol reduces bone remodelling and reciprocally affects differentiation and apoptosis of osteoblasts and osteoclasts, thereby reducing bone resorption and increasing bone formation. These effects are at least in part mediated through WNT/β-catenin signalling pathway and RANK/RANKL/OPG system. Estrogen deficiency is thus considered to be one of the major causes of postmenopausal bone loss in women and age-related bone loss in both sexes^3^. In addition, oxidative stress has been associated with decreased osteoblast and osteoclast numbers, decreased bone formation rate and increased osteoblast and osteocyte apoptosis. Reactive oxygen species (ROS) were shown to inhibit WNT/β-catenin signalling pathway in osteoblasts and increase production of adipocytes. In addition, age-related decline in estrogen levels substantially decreases defence against oxidative stress in bone thus further increasing bone resorption^4^. Another important aspect of bone health and homeostasis is angiogenic-osteogenic coupling. Hypoxia inducible factor 1α (HIF1α) signalling pathway and its target gene vascular endothelial growth factor A (*VEGFA)* are two of the most critical regulators of this coupling and are both substantially affected by hypoxia. Hypoxia has been associated with the inhibition of growth and differentiation of osteoblasts while strongly inducing osteoclast formation^5^. Conversely, selective activation of the HIF1α signalling pathway in osteoblasts showed protection from ovariectomy induced bone loss together with improved bone vascularity and angiogenesis. Furthermore, 17β-estradiol has been associated with HIF1α signalling pathway and modulation of angiogenesis^6,7^. This further underlines the importance of oxygen tension and HIF1α signalling pathway in osteogenesis.

Since the influence of estrogens, oxidative stress and hypoxia on the epigenetic mechanisms in bone is not elucidated yet the aim of our study was to identify which chromatin-modifying enzymes are substantially influenced by these factors and could be involved in the pathology of postmenopausal osteoporosis (PMO) and osteoarthritis (OA). We first determined the gene expression profile of a panel of 48 selected genes in a human osteoblast cell line HOS treated with 17β-estradiol, hydrogen peroxide and hypoxia mimetic deferoxamine (DFO). Next, the most exciting genes from the *in vitro* study were measured in human bone tissue samples of PMO and OA patients and controls. Their expression was correlated with clinically relevant phenotype data like bone mineral density (BMD), micro computed tomography (µCT) and bone histomorphometric (BHM) measurements as well as the indicators of oxidative stress, RANK/RANKL/OPG system and angiogenesis. Finally, the impact of H_2_O_2_ on the DNA methylation and histone acetylation state of HOS cells was evaluated for the highly relevant receptor activator of NF-κB ligand *(RANKL)* and osteoprotegerin (*OPG)* genes.

## Results

### Hydrogen peroxide and deferoxamine induce oxidative stress and hypoxic response in HOS cells

To study the influence of 17β-estradiol, H_2_O_2_ and DFO on chromatin-modifying enzymes’ gene expression in osteoblasts we first prepared a suitable experimental *in vitro* model using HOS cells. Since estrogen receptor α (ERα) was surprisingly completely absent HOS cells were transfected with plasmid pCMV-ESR1. The efficiency of transfection was controlled with western blot and qPCR (see Supplementary Figs S1 and S2). Based on the results of the metabolic activity assay we decided to use 500 μM H_2_O_2_ and 20 µM DFO in gene expression experiments either alone or as co-treatment with 17β-estradiol which however had no protective effect (see Supplementary Fig. S3).

**Figure 1:**
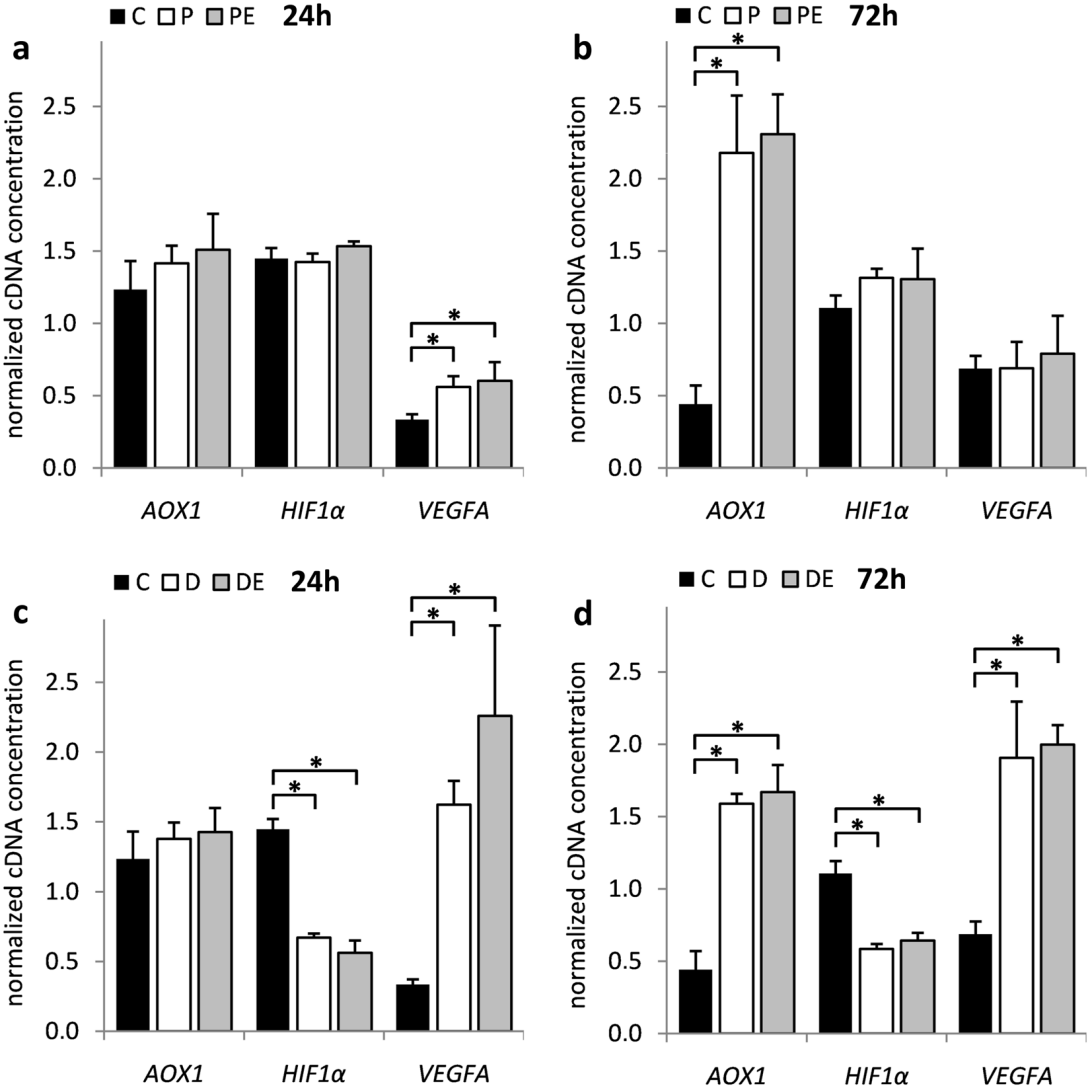
The influence of H_2_O_2_ and DFO on *AOX1*, *HIF1α* and *VEGFA* gene expression in HOS cells. HOS cells were transfected with pCMV-ESR1 and treated with 500 μM H_2_O_2_ – P, 20 µM DFO – D or vehicle control – C in the presence – PE, DE or absence of 10 nM 17β-estradiol for (**a**,**c**) 24 and (**b**,**d**) 72 hours. Values are presented as mean ± SD of normalized cDNA concentrations (n = 3). * denotes p ≤ 0.05 as compared to the vehicle control. DFO, deferoxamine.

Both, 72-hour H_2_O_2_ treatment (Fig. 1b) and DFO exposure (Fig. 1d) induced oxidative stress in HOS cells, as evidenced by a significant increase in aldehyde oxidase 1 (*AOX1*) gene expression (*p* = 5.85 × 10^−4^ and 9.68 × 10^−5^, respectively)^8^. As expected, DFO also established hypoxic response in HOS cells (Fig. 1c,d). Induction of *VEGFA* (*p* = 1.17 × 10^−2^ and 1.55 × 10^−3^, respectively) indicated transcriptional activity of HIF1α protein which is associated with its cellular accumulation and is a characteristic of hypoxia exposure. *VEGFA* (*p* = 3.56 × 10^−2^) expression was also transiently induced by H_2_O_2_ (Fig. 1a,b) which is in line with previously published data^9^. Of note, 17β-estradiol co-treatment had no influence on HOS cells regardless of the treatment performed or gene assayed (Fig. 1).

### Hydrogen peroxide and deferoxamine but not 17β-estradiol affect expression of genes encoding chromatin-modifying enzymes in HOS cells

#### Identification of affected chromatin-modifying genes by analysing the expression profile of 48 genes

Results from the qPCR screening array are presented in Supplementary Table S1. 48 genes encoding chromatin-modifying enzymes and osteoblast lineage-associated proteins were meticulously chosen on the basis of our knowledge of bone biology and epigenetics. *HAT1*, K(lysine) acetyltransferase 5 (*KAT5*) and lysine acetyltransferase 8 (*MYST1*) from the *HAT* genes, sirtuin 1 (*SIRT1*), *SIRT6*, *HDAC6*, *HDAC7* and *HDAC9* from the *HDAC* genes, and methyl-CpG binding domain protein 1 (*MBD1*) and *DNMT3A* from DNA-methylation associated genes exhibited the most pronounced changes in expression and were therefore selected for further analysis.

#### qPCR validation analyses of selected genes

**Figure 2:**
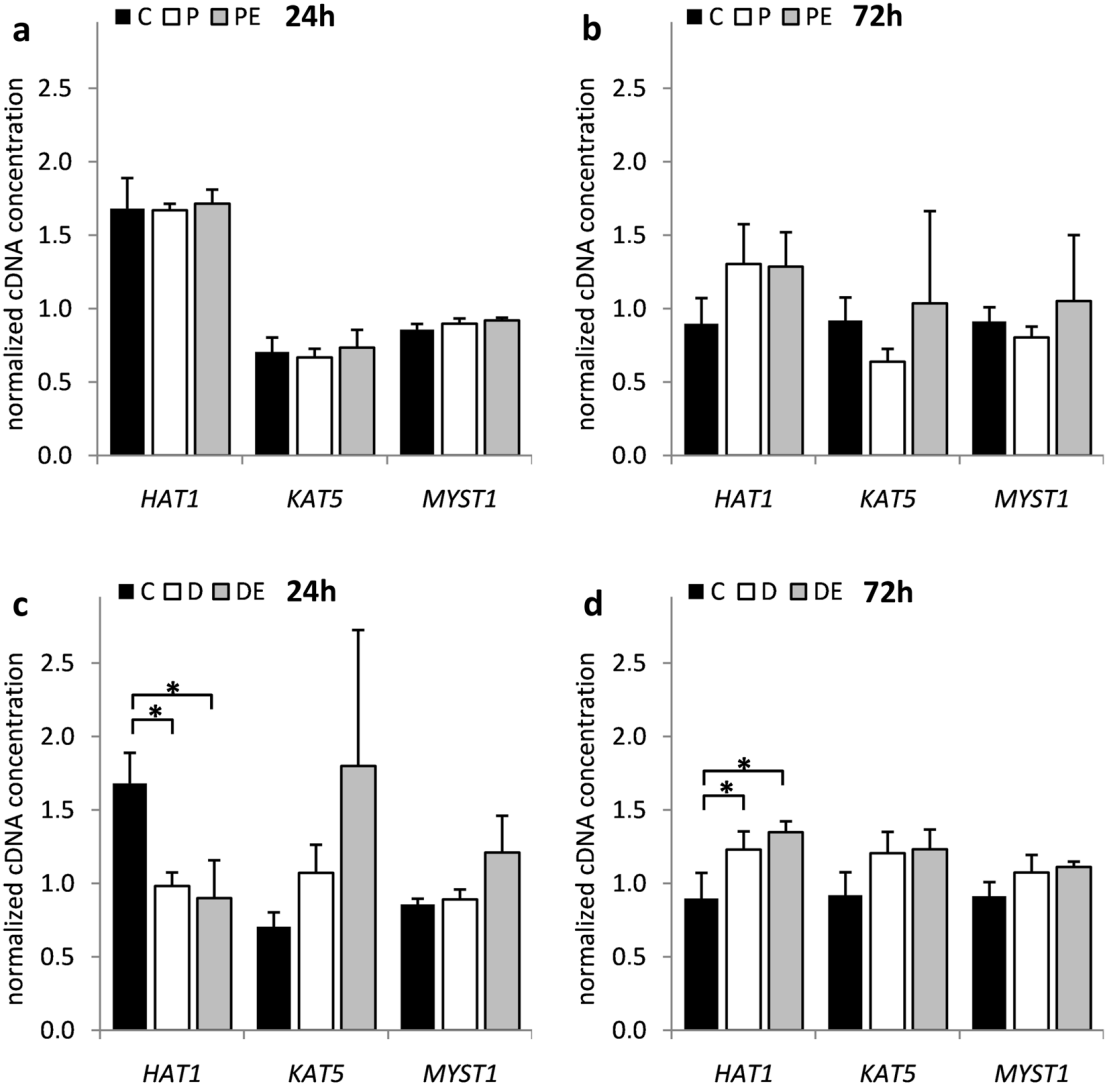
The influence of H_2_O_2_ and DFO on *HAT* gene expression in HOS cells. HOS cells were transfected with pCMV-ESR1 and treated with 500 μM H_2_O_2_ – P, 20 µM DFO – D or vehicle control – C in the presence – PE, DE or absence of 10 nM 17β-estradiol for (**a**,**c**) 24 and (**b**,**d**) 72 hours. Values are presented as mean ± SD of normalized cDNA concentrations (n = 3). * denotes p ≤ 0.05 as compared to the vehicle control. DFO, deferoxamine.

**Figure 3:**
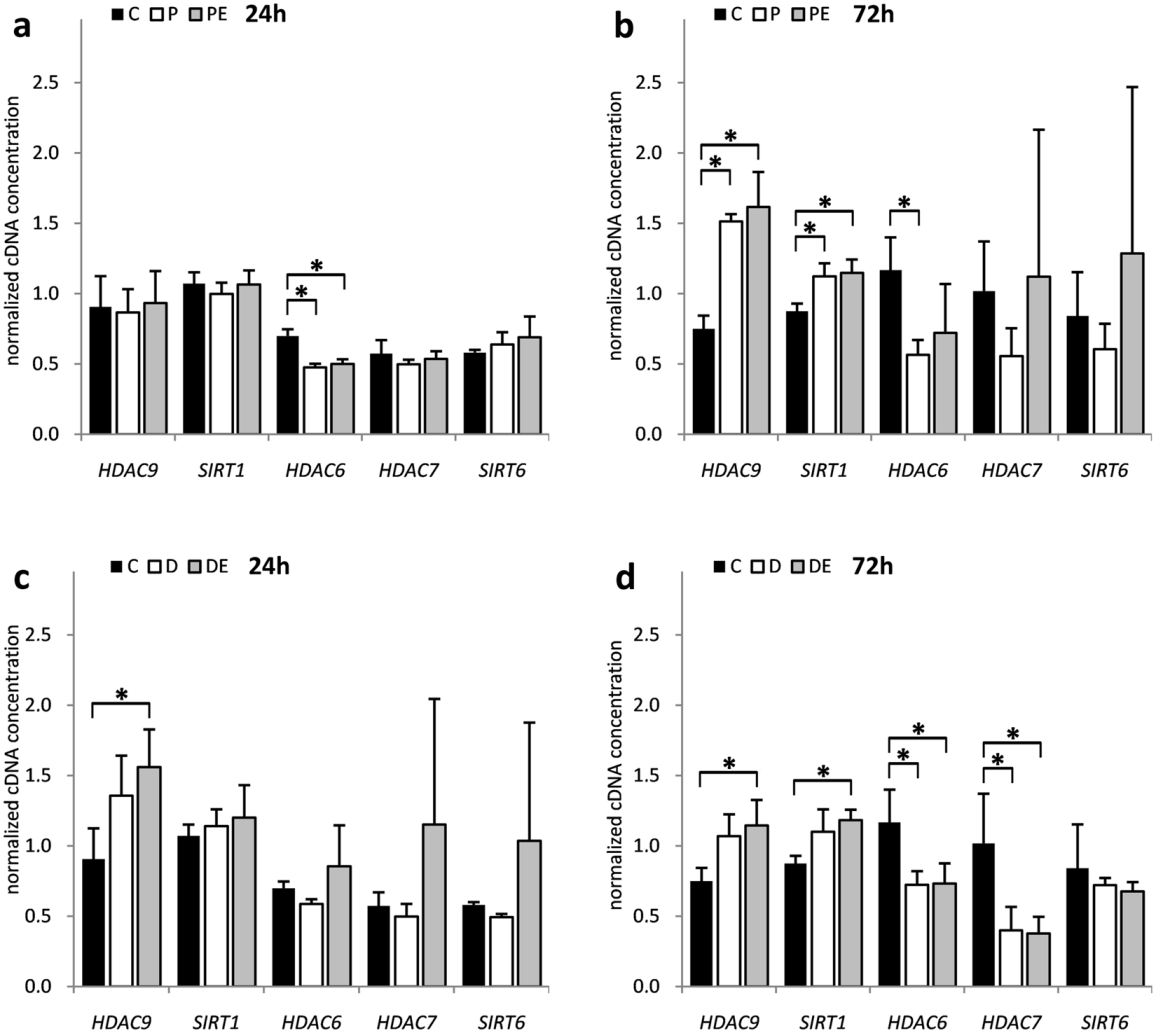
The influence of H_2_O_2_ and DFO on *HDAC* gene expression in HOS cells. HOS cells were transfected with pCMV-ESR1 and treated with 500 μM H_2_O_2_ – P, 20 µM DFO – D or vehicle control – C in the presence – PE, DE or absence of 10 nM 17β-estradiol for (**a**, **c**) 24 and (**b**, **d**) 72 hours. Values are presented as mean ± SD of normalized cDNA concentrations (n = 3). * denotes p ≤ 0.05 as compared to the vehicle control. DFO, deferoxamine.

**Figure 4:**
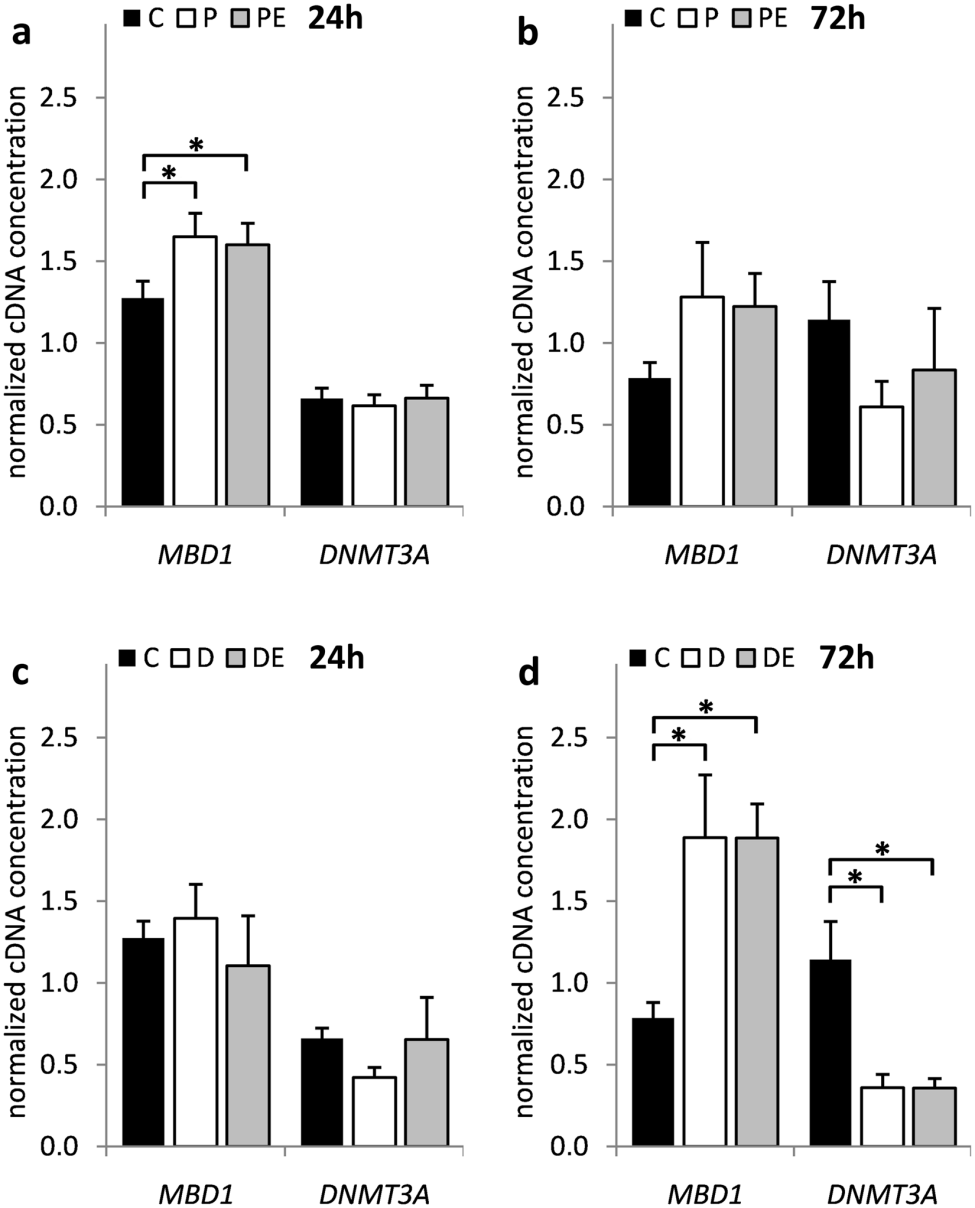
The influence of H_2_O_2_ and DFO on gene expression of enzymes associated with DNA methylation in HOS cells. HOS cells were transfected with pCMV-ESR1 and treated with 500 μM H_2_O_2_ – P, 20 µM DFO – D or vehicle control – C in the presence – PE, DE or absence of 10 nM 17β-estradiol for (**a**,**c**) 24 and (**b**,**d**) 72 hours. Values are presented as mean ± SD of normalized cDNA concentrations (n = 3). * denotes p ≤ 0.05 as compared to the vehicle control. DFO, deferoxamine.

Individual qPCR analyses revealed that DFO first significantly suppressed *HAT1* expression before inducing it at the later time point (*p* = 9.23 × 10^−3^ and 3.71 × 10^−2^, respectively) (Fig. 2). In addition, H_2_O_2_ and DFO treatment significantly suppressed the expression of *HDAC6* (*p* = 6.21 × 10^−4^, 4.36 × 10^−2^ and 3.09 × 10^−2^, respectively) and *HDAC7* (*p* = 3.21 × 10^−2^) while inducing the expression of *HDAC9* (*p* = 1.79 × 10^−3^) and *SIRT1* (*p* = 1.99 × 10^−2^) genes at different time points (Fig. 3). While *MBD1* (*p* = 2.11 × 10^−2^ and 3.54 × 10^−3^, respectively) expression was also significantly induced by both H_2_O_2_ and DFO, only DFO showed negative effect on *DNMT3A* (*p* = 1.13 × 10^−3^) (Fig. 4). Of note, none of the genes exhibited a significant response to 17β-estradiol treatment, either alone (data not shown) or as co-treatment with H_2_O_2_ or DFO.

### Diverse expression of genes encoding chromatin-modifying enzymes in bone tissue

**Figure 5:**
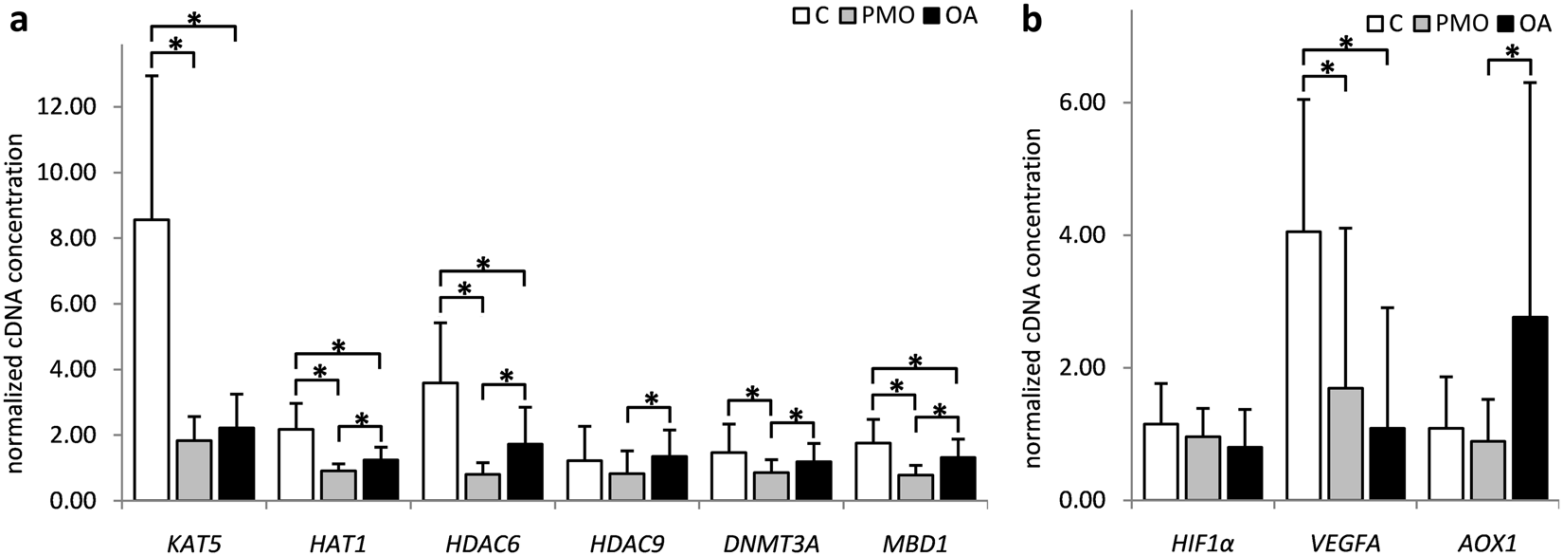
Differences in *KAT5*, *HAT1*, *HDAC6*, *HDAC9*, *DNMT3A*, *MBD1*, *HIF1α*, *VEGFA and AOX1* gene expression in bone tissue samples of controls – C, patients with postmenopausal osteoporosis – PMO and patients with osteoarthritis – OA. Values are presented as mean ± SD of normalized cDNA concentrations. *denotes p ≤ 0.05.

The selection of genes was further refined to *HAT1*, *KAT5*, *HDAC6*, *HDAC9*, *MBD1* and *DNMT3A* for qPCR analysis in bone tissue. Gene expression levels were generally the highest in control cases and the lowest in PMO patients. More specifically, expression of *KAT5* was significantly higher in the control group as compared to both patient groups (*p* = 3.08 × 10^−7^ and 1.16 × 10^−6^, respectively) (Fig. 5a). Expression of *HAT1* (*p* = 1.18 × 10^−5^, 1.10 × 10^−4^ and 1.69 × 10^−7^, respectively), *HDAC6* (*p* = 1.07 × 10^−5^, 3.56 × 10^−4^ and 3.05 × 10^−11^, respectively) and *MBD1* (*p* = 3.09 × 10^−7^, 2.58 × 10^−2^ and 3.41 × 10^−5^, respectively) differed significantly between all three tested groups, wherein control samples again exhibited the highest levels of expression followed by OA patients and then PMO patients. *DNMT3A* expression was significantly lower in PMO patients than in either control samples or OA patients (*p* = 2.99 × 10^−2^ and 2.95 × 10^−2^, respectively). Similarly, *HDAC9* expression was also significantly lower in PMO patients as compared to OA patients (*p* = 1.16 × 10^−5^).

We additionally looked at the expression of *HIF1α*, *VEGFA* and *AOX1* in the same bone tissue samples. Expression of *VEGFA* was significantly higher in controls as compared to both patient groups (*p* = 1.77 × 10^−4^ and 3.60 × 10^−6^, respectively) (Fig. 5b). Conversely, *AOX1* exhibited significantly higher expression in OA patients than in PMO patients (*p* = 4.06 × 10^−4^). Expression of *HIF1α* did not differ between the tested groups.

### Correlation between expression of genes encoding chromatin-modifying enzymes in bone tissue and the parameters of bone quantity and quality

Gene expression of all chromatin-modifying enzymes analysed in bone tissue apart from *KAT5* showed a substantial positive correlation with BMD at least at one of the measured sites, with *MBD1* correlating substantially at all three (Table 1). *HAT1*, *HDAC6* and *KAT5* exhibited a substantial positive correlation with trabecular number (Tb.N) and concurrently a substantial negative correlation with trabecular separation (Tb.Sp) indicating a strong association of these three genes with bone microarchitecture (Table 1). Contrary to BHM values, bone quality and quantity parameters determined by µCT measurements had not revealed any substantial correlations with gene expression data. We detected however the strongest correlation between the expression of *OPG* and *AOX1* genes and *HAT1*, *HDAC6*, *HDAC9*, *DNMTA3A* and *MBD1*, the same genes that also substantially correlated with BMD. Correlation coefficients between 0.434 and 0.726 with *p* values between 1.02 × 10^−5^ and 6,13 × 10^−17^ provided a strong argument for a close relationship between these chromatin-modifying enzymes and indicators of bone formation and oxidative stress response (Table 1). Interestingly, expression of *RANKL* correlated with altogether different genes from our set than *OPG*, namely negatively with *KAT5* and positively with *HIF1α* (Table 1).

**Table 1:** Correlation analysis of genes analysed in bone tissue and bone quality and quantity parameters.

	*KAT5*	*HAT1*	*HDAC6*	*HDAC9*	*DNMT3A*	*MBD1*	*HIF1α*	*VEGFA*	*AOX1*
Hip BMD	0.037	0.361^a^	0.394^a^	0.221	0.259	0.445^a^	0.002	0.225	0.180
Fn BMD	0.121	0.410^a^	0.405^a^	0.307^a^	0.363^a^	0.476^a^	−0.088	0.222	0.179
Ls BMD	−0.093	0.171	0.250	0.140	0.240	0.306^a^	−0.119	0.084	0.114
BV/TV (µCT)	0.238	0.151	0.094	−0.101	−0.011	0.083	−0.207	0.045	0.044
BS/BV (µCT)	−0.137	0.142	0.113	0.164	0.249	0.241	0.208	0.100	0.116
BS/TV (µCT)	0.263	0.258	0.215	−0.051	0.127	0.224	−0.187	0.090	0.101
Tb.Th (µCT)	0.048	−0.162	−0.137	−0.122	−0.203	−0.248	−0.067	−0.052	−0.108
Tb.Sp (µCT)	−0.142	−0.295	−0.203	−0.083	−0.269	−0.294	0.114	−0.035	0.024
Tb.N (µCT)	0.258	0.247	0.202	−0.120	0.084	0.217	−0.223	0.123	0.114
BV/TV (BHM)	0.217	0.278	0.335	0.014	0.067	0.201	−0.161	0.078	0.163
BS/BV (BHM)	−0.083	−0.091	−0.098	0.034	0.174	0.043	0.264	0.103	0.058
BS/TV (BHM)	0.346	0.449	0.525^a^	0.077	0.276	0.418	−0.037	0.277	0.312
Tb.Th (BHM)	−0.049	−0.044	−0.044	−0.036	−0.206	−0.136	−0.311	−0.224	−0.022
Tb.Sp (BHM)	−0.472^a^	−0.546^a^	−0.599^a^	−0.026	−0.232	−0.430	−0.010	−0.379	−0.190
Tb.N (BHM)	0.459^a^	0.505^a^	0.588^a^	0.081	0.268	0.434	0.048	0.390	0.266
*RANKL*	−0.465^a^	−0.106	−0.217	−0.088	0.101	0.015	0.623^a^	0.245	0.149
*OPG*	−0.118	0.488^a^	0.570^a^	0.453^a^	0.537^a^	0.647^a^	0.223	0.303^a^	0.626^a^
*AOX1*	0.023	0.434^a^	0.479^a^	0.726^a^	0.502^a^	0.526^a^	0.121	−0.112	/
*HIF1α*	−0.172	0.066	−0.089	−0.008	0.030	0.073	/	/	/
*VEGFA*	0.137	0.313^a^	0.209	−0.211	0.083	0.374^a^	0.423^a^	/	/

Values represent correlation coefficients of Spearman’s rho analysis. ^a^Denotes p ≤ 0.009 (adjusted for multiple testing using false discovery rate method). BS, bone surface; BV, bone volume; Fn, femoral neck; Ls, lumbar spine; Tb.N, trabecular number; Tb.Sp, trabecular separation; Tb.Th, trabecular thickness; TV, tissue volume.

### Diverse effects of hydrogen peroxide and deferoxamine on WNT/β-catenin signalling pathway and RANK/RANKL/OPG system in HOS cells

**Figure 6:**
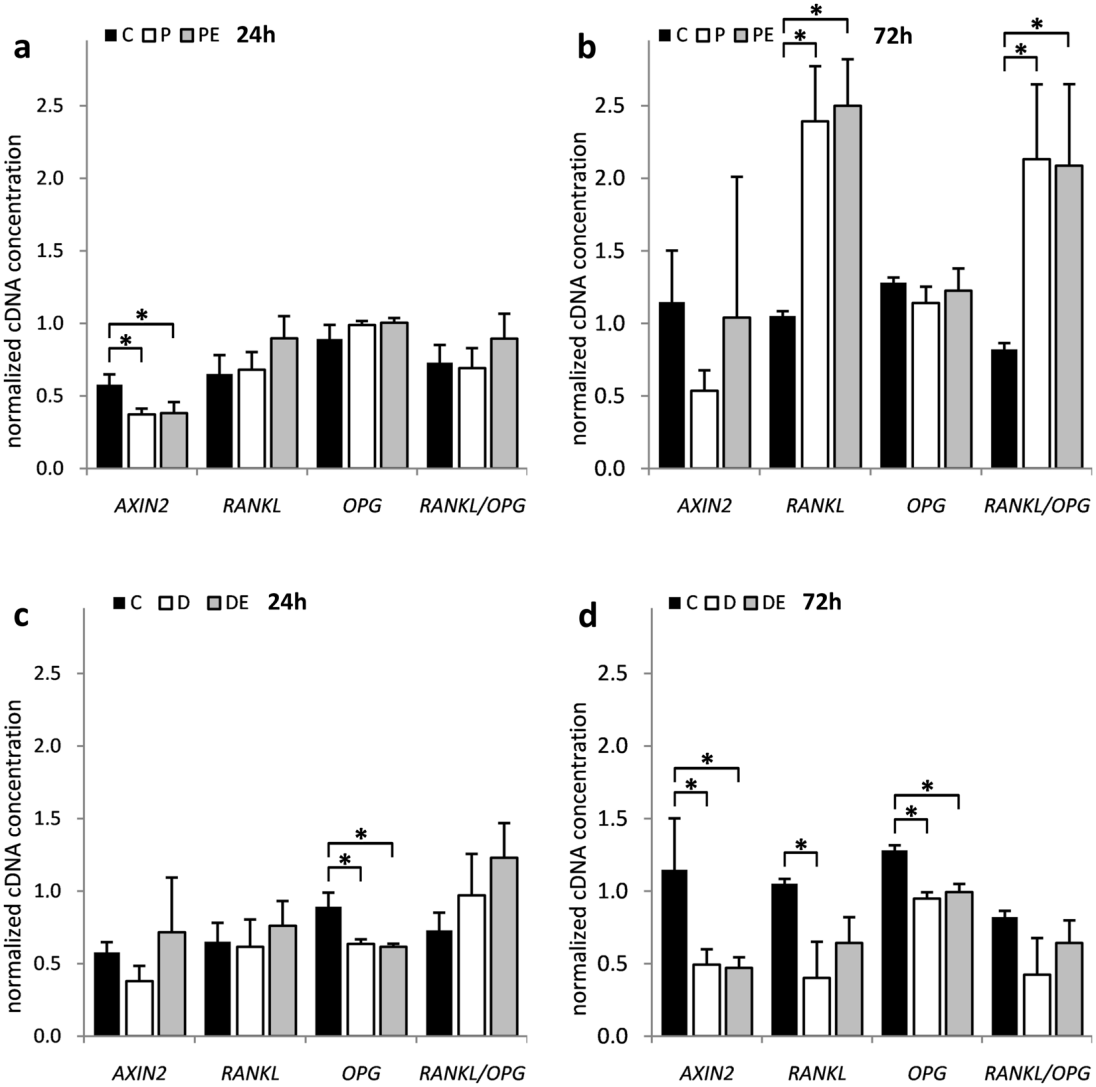
The influence of H_2_O_2_ and DFO on *AXIN2*, *RANKL* and *OPG* gene expression and *RANKL/OPG* ratio in HOS cells. HOS cells were transfected with pCMV-ESR1 and treated with 500 μM H_2_O_2_ – P, 20 µM DFO – D or vehicle control – C in the presence – PE, DE or absence of 10 nM 17β-estradiol for (**a**,**c**) 24 and (**b**,**d**) 72 hours. Values are presented as mean ± SD of normalized cDNA concentrations (n = 3). * denotes p ≤ 0.05 as compared to the vehicle control. DFO, deferoxamine.

WNT/β-catenin signalling pathway was significantly suppressed in HOS cells after a 24-hour exposure to H_2_O_2_ and a 72-hour exposure to DFO, as indicated by changes in *AXIN2* expression (*p* = 1.50 × 10^−2^ and 1.89 × 10^−2^, respectively) (Fig. 6). Conversely, *RANKL* and *OPG* displayed much more diverse changes. H_2_O_2_ significantly induced *RANKL* (*p* = 2.29 × 10^−3^) expression after 72 hours of treatment which resulted in significantly increased *RANKL/OPG* ratio (*p* = 1.96 × 10^−2^). DFO, on the other hand, significantly suppressed *RANKL* (*p* = 7.78 × 10^−3^) and *OPG* expression (*p* = 3.84 × 10^−3^ and 2.49 × 10^−4^), however no significant changes in the *RANKL/OPG* ratio occurred (Fig. 6). Again, 17β-estradiol showed no significant impact on expression of selected genes either alone (data not shown) or as a co-treatment (Fig. 6).

### Effect of hydrogen peroxide on DNA methylation levels at *RANKL* and *OPG* promoters in HOS cells

**Figure 7:**
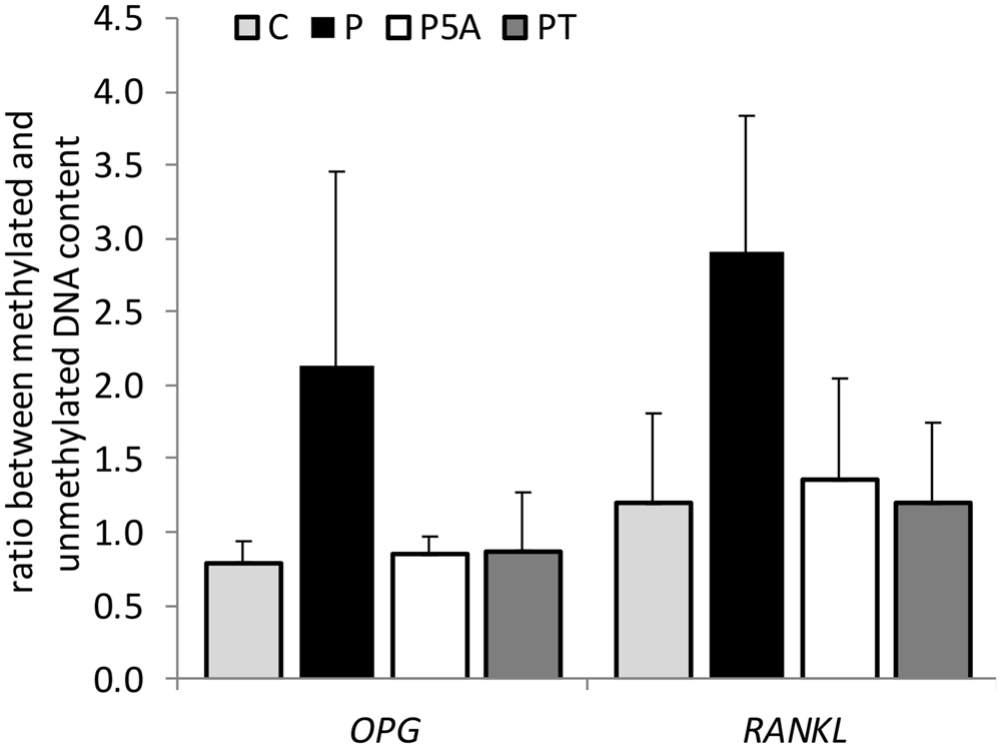
The influence of H_2_O_2_, 5-azacytidine and tempol on the ratio between methylated and unmethylated DNA at *OPG* and *RANKL* promoters in HOS cells. HOS cells were treated for 24 hours with vehicle control – C, 500 μM H_2_O_2_ alone – P or in the presence of 2.5 μM 5-azacytidine – P5A or 200 μM tempol – PT. Values are presented as mean ± SD of the ratio between methylated and unmethylated DNA content (n = 3).

We examined two CpG islands within *RANKL* and *OPG* promoters, previously shown to influence their transcription^10^. In both cases a clear positive trend was evident after 24-hour exposure to H_2_O_2_ (Fig. 7). Although not significant, the fact that this increase in DNA methylation levels was prevented by co-treatment with DNMT inhibitor 5-azacytidine and antioxidant tempol (Fig. 7), indicates that the observed changes in DNA methylation were indeed mediated by oxidative stress. However; there were no such changes when a lower concentration of H_2_O_2_ was used (see Supplementary Fig. S4).

### Effect of hydrogen peroxide on histone H3 acetylation in *RANKL* and *OPG* genes in HOS cells

**Figure 8:**
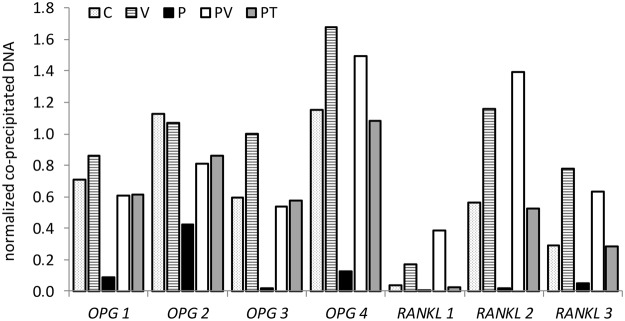
The influence of H_2_O_2_, vorinostat and tempol on the levels of histone H3 acetylation at four *OPG* and three *RANKL* sites in HOS cells. HOS cells were treated for 24 hours with vehicle control – C, 0.625 μM vorinostat – V, 500 μM H_2_O_2_ alone – P or in the presence of 0.625 μM vorinostat – PV or 200 μM tempol – PT. Values from a single experiment are shown as normalized co-precipitated DNA concentrations (n = 1).

We chose four sites in the *OPG* and three in the *RANKL* gene, previously shown to interact with histones^11^. 24-hour treatment with H_2_O_2_ markedly reduced or almost completely prevented acetylation of H3 at all sites analysed (Figs 8 and S5). Co-treatment with HDAC inhibitor vorinostat and tempol efficiently prevented H_2_O_2_-induced acetylation changes, again indicating that oxidative stress was the driver of the observed epigenetic changes (Fig. 8).

**Figure 9:**
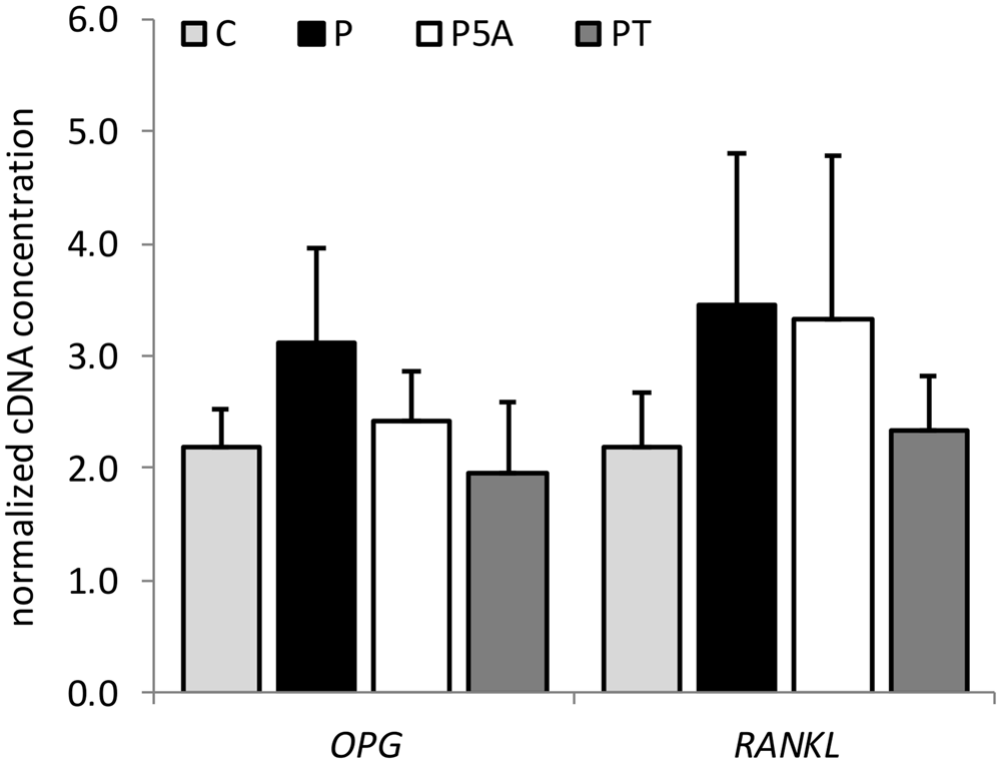
The influence of H_2_O_2_, 5-azacytidine and tempol on *OPG* and *RANKL* gene expression in HOS cells. HOS cells were treated for 24 hours with vehicle control – C, 500 μM H_2_O_2_ alone – P or in the presence of 2.5 μM 5-azacytidine – P5A or 200 μM tempol – PT. Values are presented as mean ± SD of normalized cDNA concentrations (n = 3).

Despite the observed DNA methylation and H3 acetylation changes, *RANKL* and *OPG* expression levels were not significantly affected by H_2_O_2_ at this time point (Fig. 9). This together with the lack of effect from the 5-azacytidine and tempol treatment indicates that there are additional factors co-regulating expression of these two genes under the influence of oxidative stress.

## Discussion

Epigenetic mechanisms have been recognized as important regulators of bone health and disease; however little is known about the factors and the enzymes responsible for epigenetic changes in bone cells and tissue. In this study, we show that genes encoding chromatin-modifying enzymes are significantly affected by oxidative stress and hypoxia mimetic in HOS cells. Moreover, we are the first to reveal that chromatin-modifying enzymes are differentially expressed in bone tissue samples from PMO and OA patients, and control samples and significantly correlated with BMD and BHM parameters making them an important consideration in the study of age-related bone diseases.

We used three stimuli with an important role in bone biology, 17β-estrogen, oxidative stress and hypoxia to evaluate their influence on epigenetic regulation of bone remodelling in HOS cells before validating the significance of the affected chromatin-modifying enzymes on bone tissue samples from PMO and OA patients and healthy controls. Three genes in particular, i.e. *HAT1*, *HDAC6* and *MBD1*, exhibited the biggest variation between our three patient groups. This together with their significant correlation with a number of measured bone tissue phenotype parameters indicates that higher expression of *HAT1*, *HDAC6* and *MBD1* is associated with superior bone quantity as well as quality of the bone tissue having a more favourable trabecular structure. The phenotype of our bone tissue samples was characterized very thoroughly using most reliable parameters for bone quantity (BMD) as well as bone quality (µCT and BHM analysis). The latter are rarely available thus importantly contributing to the relevance of our study. Furthermore, *HAT1*, *HDAC6* and *MBD1* positive correlation with *OPG* and *VEGFA* expression indicate that their positive effect on bone could be mediated through their impact on the RANK/RANKL/OPG system and angiogenesis either by directly regulating the expression of *OPG* and *VEGF* or indirectly by regulating the expression of other important factors upstream from these two genes. In addition, their positive correlation with *AOX1* expression clearly shows that oxidative stress present in bone tissue has a significant impact on the expression of chromatin-modifying enzymes which thus represent an additional mechanism by which oxidative stress affects bone metabolism. Limited information is available so far regarding *HAT1*, *HDAC6* and *MBD1* role in bone tissue. HAT1 has been reported to have a role in embryonic bone development, while HDAC6 could affect bone tissue through its involvement in angiogenesis and RUNX2 interaction^12–14^.

Interestingly, *HDAC9* and *DNMT3A* gene expressions were significantly lower in PMO than in OA patients, while their expressions in OA patients didn’t differ from that in controls. This indicates that their expressions are aberrant only in pathological changes associated with PMO and not OA thus linking their suppression to bone loss. In addition, their expression also significantly correlated with parameters of bone quantity as well as *OPG* and *AOX1* linking them to the RANK/RANKL/OPG system and oxidative stress response in a similar way to the already described *HAT1*, *HDAC6* and *MBD1*. Based on published data HDAC9 could also potentially impact the skeleton by directing the differentiation of mesenchymal stem cells towards osteoblasts instead of adipocytes while our study is one of the first pointing towards a significant role of DNMT3A in bone tissue^15,16^.

Contrary to the described five genes *KAT5* expression did not differ between the two patient groups however it was significantly lower than in controls. This indicates that there occur certain changes in epigenetic regulation in bone tissue that could be involved in the pathogenesis of both diseases. Interestingly, our results revealed a negative correlation between *KAT5* and *RANKL* expression also linking it to the RANK/RANKL/OPG system and confirming its regulatory potential of bone remodelling similar to that of the already described five genes.

We also observed a strong expression of *VEGFA* in normal bone tissue which would indicate good vascularization; however, there was no correlation between *VEGFA* expression and parameters of bone quality and quantity. Moreover, expression of *VEGFA* was similar in OA and PMO patients even though they have significantly different BMD and bone quality^17^. On the other hand, there was significant correlation between *VEGFA* expression and expression of *HAT1* and *MBD1*, suggesting there is a certain relationship between angiogenesis and epigenetic mechanisms in bone tissue.

Even though oxidative stress has been primarily associated with bone loss in PMO patients, our results revealed the highest expression of *AOX1* in OA bone tissue^18^. Oxidative stress is in fact a relevant part of pathogenesis of OA and is likely to be involved in cartilage destruction and bone cyst formation^19^. In line with that, *HDAC9*, shown to be induced by oxidative stress in our *in vitro* model, also exhibited the highest expression in OA bone.

Finally, we have shown that changes in gene expression of chromatin-modifying enzymes are accompanied by alterations of DNA methylation and histone acetylation levels in HOS cells when exposed to oxidative stress. Using *RANKL* and *OPG* as an example indicated, that oxidative stress increased DNA methylation and decreased histone acetylation at these two genes. While this could cause downregulation of target genes, additional regulatory mechanisms seem to be induced by oxidative stress representing a higher level of regulation. This however does not diminish the relevance of our results since regulation of bone remodelling is highly complex and involves numerous other factors that could be epigenetically regulated beside RANKL an OPG. In addition, involvement of oxidative stress-induced epigenetic changes has been reported for diseases such as chronic obstructive pulmonary disease^20^, ageing^21^ and cancer^22,23^.

There are some limitations to our study. Due to a very restricted availability and challenging collection of bone tissue samples, we were not able to completely match the number and gender structure of our autopsy control group of patients to the PMO and OA groups. However, because we didn’t observe any systemic bias in gene expression results for this patient group we think that having three groups of patients spanning a wide range of bone quality and quantity phenotypes adds an important validity to our study that is often missing in other studies using human bone tissue samples. The other limitation of our study is the use of HOS cells to study the effect of estrogen signalling. There are several reports in the literature describing ERα mRNA and protein expression in this cell line; however, we were unable to detect neither of them despite testing two different batches of HOS cells obtained from the ATCC^24–26^. In order to overcome this problem, we finally decided to transfect them with a ERα-carrying plasmid.

## Conclusion

In conclusion we have translated our *in vitro* observations of hypoxia and oxidative stress induced expression changes in chromatin-modifying enzymes to clinical samples demonstrating that *HAT1*, *KAT5*, *HDAC6*, *MBD1* and *DNMT3A* expression was both significantly downregulated in PMO and OA. Moreover, higher expression of *HAT1*, *HDAC6* and *MBD1* was associated with superior quantity and quality of the bone tissue having a more favourable trabecular structure. This is the first report showing the importance of these enzymes in the maintenance of bone homeostasis and their possible involvement in the pathology of PMO and OA and certain other bone disorders.

## Methods

### Patients

The patient cohort was described and examined by Dragojevič *et al*.^27^. Briefly, our study included 96 patients, 43 with a fragility fracture of the hip due to PMO, 41 with primary end-stage OA of the hip and 14 autopsy controls without any signs or history of disease or medication use known to affect bone or mineral metabolism (see Supplementary Table S2). All patients apart from the controls underwent hip arthroplastic surgery during which trabecular bone tissue samples from the intertrochanteric region of the proximal femur were collected. PMO was diagnosed based on a non-traumatic, low-energy hip fracture, while OA was diagnosed by clinical and radiographic criteria according to the Harris hip score. All PMO patients were submitted to arthroplasty within 24 h following femoral neck fracture whereby the site of sample collection was located away from the fracture site. Patients with any diseases (other than PMO or primary OA of the hip) or medication use known to influence bone quality or mineral metabolism were excluded from the study. BMD measurements of the contralateral hip and lumbar spine were performed only in PMO and OA patients as described previously^27^. μCT and BHM analysis was carried out on bone tissue samples from a subset of patients (11 PMO, 11 OA and 12 autopsy controls) as described previously^17^. Briefly, bone samples were scanned using a Skyscan 1,172 mCT scanner (Skyscan, Aartselaar, Belgium) at two resolutions (10 and 20 mm voxel size). After scanning, undecalcified bone samples were embedded in methylmethacrylate, stained with von Kossa van Giesson. A semi-automated program, developed by dr. van’t Hof was used^28^. Total RNA was isolated from bone tissue samples of all patients and reverse transcribed as described previously^27^. Patients were grouped on the basis of their precisely determined bone phenotype. Due to low numbers of male patients and high phenotypic homogeneity within the groups we restrained from further gender-based subgrouping. The study was approved by Komisija republike Slovenije za medicinsko etiko and a signed informed consent form was obtained from each patient. All experiments were carried out in accordance with the approved guidelines and related regulations.

### RNA isolation and reverse transcription

Human osteosarcoma cell line HOS TE-85 (female, Caucasian, 13 years old) was obtained from the American Type Culture Collection (ATCC, Manassas, VA, USA) and maintained as described previously^29^. Cells were seeded in T25 cell culture flasks at a density of approximately 12000 and 7000 cells/cm^2^ depending on the duration of treatment. Transient transfection was performed with plasmid pCMV-ESR1 (Sino biological, Beijing, China) and X-tremeGENE HP DNA transfection reagent (Roche Applied Science, Mannheim, Germany) according to manufacturer’s instructions; however the amount of plasmid DNA was reduced because of high ERα protein levels post transfection. Concomitantly cells were treated with 10 nM 17β-estradiol (Sigma-Aldrich, St. Louis, MO, USA) or vehicle control for 24 hours. After transfection and pre-treatment cells were exposed to 500 μM H_2_O_2_ (Sigma-Aldrich, St. Louis, MO, USA), 20 µM DFO (Sigma-Aldrich, St. Louis, MO, USA) or vehicle control. Following 24 and 72 hours of treatment cells were lysed directly in the cell culture flask and total RNA was isolated using miRNeasy Mini Kit (Qiagen, Hilden, Germany) according to manufacturer’s instructions. RNA integrity was checked using 2100 Bioanalyzer (Agilent Technologies, Palo Alto, CA, USA). 2 µg of RNA were reverse transcribed with Transcriptor First Strand cDNA Synthesis Kit (Roche Applied Science, Mannheim, Germany) according to manufacturer’s instructions.

### Expression profiling using qPCR Array

A RealTime ready custom panel 384 – 48 (Roche Applied Science, Mannheim, Germany) was designed and used for gene expression profiling as described previously^29^. A complete list and short description of the 48 selected genes is included in Supplementary Table S3. The 2^−ΔΔCt^ method was used for analysis of relative gene expression data^30^. Ribosomal protein, large, P0 (*RPLP0*) and lysine acetyltransferase 6B *(MYST4)* were selected as reference genes based on their stability of expression determined with NormFinder software^31^.

### Individual qPCR analyses

In addition to the genes selected in the screening step (*KAT5*, *HAT1*, *MYST1*, *HDAC6*, *HDAC7*, *HDAC9*, *SIRT1*, *SIRT6*, *DNMT3A* and *MBD1*), *AOX1*, *HIF1α*, *VEGFA*, *AXIN2*, *RANKL*, *OPG*, *ERα*, *ERβ* and G protein-coupled estrogen receptor 1 (*GPER1*) were included in the analysis. Primer pairs (see Supplementary Table S4) were designed using Primer-BLAST on-line tool or obtained from the literature and used with SYBR Select Master Mix (Applied Biosystems, Foster City, CA, USA) according to manufacturer’s instructions. 20 ng of cDNA was used per reaction, except for *RANKL* and *ERβ* analysis where 40 ng was required due to low expression levels observed in preliminary tests. A cDNA dilution curve was used for quantification and geometric mean of *RPLP0* and *MYST4* expression levels for normalization. All three biological replicates were analysed and all amplifications were performed on LightCycler 480 Instrument II (Roche Applied Science, Mannheim, Germany).

A gene expression analysis of a subset of genes (*KAT5*, *HAT1*, *HDAC6*, *HDAC9*, *DNMT3A*, *MBD1*, *AOX1*, *HIF1α and VEGFA*) was also performed on RNA isolated from bone tissue samples of PMO and OA patients and controls. qPCR analysis was performed as for cell culture experiments with certain exceptions; i.e. only 15 ng of cDNA was used per reaction and *RPLP0* and glyceraldehyde-3-phosphate dehydrogenase (*GAPDH*) were used as reference genes^32^. Gene expression data on *RANKL* and *OPG* used in the correlation analysis was already published by Dragojevič *et al*.^27^.

### Statistical analysis

Differences in anthropometric parameters between patients were analysed using one-way analysis of variance (ANOVA) with Tukey’s *post hoc* test (age and BMI) or Student’s t test for independent samples (BMD and *t-score* values). Gender differences between groups were assessed using Fisher’s test. Differences in gene expression and viability of HOS cells between treated groups and controls were assessed using one-way ANOVA with Dunnett’s *post hoc* test. Gene expression data obtained on bone tissue samples that exhibited normal distribution was analysed using analysis of covariance (ANCOVA) with age, gender and BMI as covariates and Bonferroni *post hoc* test. Data that didn’t meet the assumptions for parametric tests was first analysed using the Kruskal-Wallis and then a series of Mann-Whitney U tests with a *p* value corrected according to the Bonferroni method. Spearman’s rho correlation analysis and false discovery rate method to control for multiple testing were employed to examine relationships between gene expression data and bone quality parameters. All tests were performed two-tailed with accepted alpha level of 0.05. Results with a *p* value of 0.05 or less were considered statistically significant apart from Spearman’s rho correlation analysis where *p* value of 0.009 was used. All statistical analyses were performed using SPSS Statistics software 22 (IBM, Chicago, IL, USA).

### Supplementary Methods

Detailed descriptions of quantitative methylation-specific PCR, chromatin immunoprecipitation, metabolic activity assay and western blot analysis are provided in the Supplementary Information.

## Electronic supplementary material

Supplementary information

## Data Availability

All data generated or analysed during this study are included in this published article (and its Supplementary Information) and ref.^27^.
