# Supplementary material for: Epigenetic enzymes influenced by oxidative stress and hypoxia mimetic in osteoblasts are differentially expressed in patients with osteoporosis and osteoarthritis
Source: Sci Rep. 2018 Nov 1;8:16215. doi: 10.1038/s41598-018-34255-4 (PMC6212423; doi:10.1038/s41598-018-34255-4)
Supplement: Supplementary file 1 — Supplementary information [file 41598_2018_34255_MOESM1_ESM.docx]

Supplementary Information

**Epigenetic enzymes influenced by oxidative stress and hypoxia mimetic in osteoblasts are differentially expressed in patients with osteoporosis and osteoarthritis**

Peter Vrtačnik^1^, Janja Zupan^1^, Vid Mlakar^1^, Tilen Kranjc^1^, Janja Marc^1^, Barbara Kern^1^, Barbara Ostanek^1*^

^1^Department of Clinical Biochemistry, Faculty of Pharmacy, University of Ljubljana, Aškerčeva cesta 7, SI-1000 Ljubljana, Slovenia

**Supplementary methods**

***Quantitative methylation-specific PCR***

Where indicated HOS cells where treated with 500 μM H_2_O_2_, 200 μM tempol (Sigma-Aldrich, St. Louis, MO, USA) or 2.5 μM 5-azacytidine (Sigma-Aldrich, St. Louis, MO, USA). DNA was isolated from 2x10^6^ HOS cells using Flexigene kit (Qiagen, Hilden, Germany). 1000 ng of DNA was bisulfite converted using EpiTect Fast Bisulfite Conversion kit (Qiagen, Hilden, Germany). Quantitative methylation-specific PCR (qMSP) was performed using HOT FIREPol EvaGreen Supermix (Solis Biodyne, Tartu, Estonia), 5 μl of bisulfite converted DNA and 100 nM primers specific for methylated or unmethylated regions of receptor activator of NF-κB ligand (RANKL) or osteoprotegerin (OPG) promoter regions and described previously (Supplementary Table S5).[^1^](#_ENREF_1) Glyceraldehyde-3-phosphate dehydrogenase (GAPDH) promoter region was used as a loading control. DNA concentration was calculated from the standard curve and normalized to the concentration of GAPDH promoter region. Normalized concentration was further standardized (zero-mean-unit-variance) within experimental batch to account for batch differences. The ratio between methylated and unmethylated DNA was calculated by dividing standardized concentrations.

***Chromatin immunoprecipitation***

Where indicated HOS cells where treated with 500 μM H_2_O_2_, 200 μM tempol or 0.625 μM vorinostat (Sigma-Aldrich, St. Louis, MO, USA). Chromatin immunoprecipitation (ChIP) was performed using Acetyl-Histone H3 Immunoprecipitation (ChIP) assay kit (Merck Millipore, Kenilworth, NJ, USA). 8x10^6^ HOS cells were fixed with 1% paraformaldehyde, washed and collected in ice-cold phosphate-buffered saline (PBS) containing cOmplete Mini protease inhibitors (Roche Applied Science, Mannheim, Germany). Cell pellet was lysed in 1 ml of lysis buffer and stored at -20°C until further processing. Cell lysate was sonicated using an ultrasonic probe for 13 rounds of 10 second bursts at 15 W and centrifuged. Supernatant was diluted with dilution buffer to a total volume of 2 ml and 200 µl aliquots were saved to use as loading controls. Diluted lysate was incubated with Protein A-agarose beads blocked with salmon sperm DNA for 1 h to reduce non-specific background followed by an overnight incubation with 10 μl of anti-acetyl-histone H3 antibody at 4°C. Immune complexes were captured to protein A-agarose beads blocked with salmon sperm DNA with a 4-hour incubation at 4°C followed by 15-minute washes with the following buffers: low salt wash buffer, high salt wash buffer, LiCl wash buffer and TE buffer. Histones crosslinked with DNA were eluted twice with 100 μl of freshly prepared elution buffer (1% SDS, 0.1M NaHCO_3_). 8 µl of 5M NaCl was added to the combined eluates and incubated overnight at 65°C. Decrosslinked eluates were incubated with RNAse A (Omega Bio-tek, Norcross, GA, USA) at 37°C for 15 minutes, followed by proteinase K (VWR, Radnor, PA, USA) digestion at 55°C for 15 minutes. DNA was purified using PCR purification columns (Solarbio, Beijing, China).

Fragments from the promoter regions of *RANKL* and *OPG* genes were quantified with qPCR using HOT FIREPol EvaGreen SuperMix, as described above. Primers for the four regions in *OPG* and the three regions in *RANKL* gene were designed using Primer-BLAST (Supplementary Table S4). DNA concentration was calculated from the standard curve and normalized to the loading control.

***Metabolic activity assay***

HOS cells were seeded in 96-well cell culture plates at a density of approximately 4000 and 2000 cells per well depending on the duration of treatment. Following transient transfection with pCMV-ESR1 and 24-hour pre-treatment with 17β-estradiol or vehicle control the cells were exposed to increasing concentrations of H_2_O_2_, deferoxamine (DFO) or vehicle control. 24 and 72 hours after exposure the number of viable cells was determined with the use of CellTiter 96® Aqueous One Solution Cell Proliferation Assay (Promega, Madison, WI, USA) according to manufacturer’s instructions. The experiment was repeated three times.

***Western blot analysis***

Proteins were isolated from the same cell lysates as RNA. After RNA isolation and DNA removal proteins were precipitated from the organic phase with 2-propanol (Merck Millipore, Kenilworth, NJ, USA) and washed with 0.3 M guanidine (Sigma-Aldrich, St. Louis, MO, USA) solution and ethanol alone. Protein pellet was air-dried and dissolved in 1 % sodium dodecylsulfate (SDS) (Promega, Madison, WI, USA). Protein concentrations were determined using the DC protein assay (Bio-Rad Laboratories, Hercules, CA, USA). Up to 45 μg of protein samples were resolved with SDS-polyacrylamide gel electrophoresis and transferred to nitrocellulose membranes using the iBlot gel transfer device (Invitrogen, Carlsbad, CA, USA). Primary anti-ERα (D-12) (Santa Cruz Biotechnology, Inc., Santa Cruz, CA, USA) and anti-β actin (Sigma-Aldrich, St. Louis, MO, USA) and secondary horseradish peroxidase-coupled anti-mouse antibodies (Merck Millipore, Kenilworth, NJ, USA) were used. Bands were detected using the SuperSignal West Femto substrate (Pierce Biotechnology, Rockford, IL, USA) and the G:Box imaging system (Syngene, Cambridge, UK).

|  |  | 24 h | | | 72 h | | |  |
| --- | --- | --- | --- | --- | --- | --- | --- | --- |
|  | Gene | 17β-estradiol  (log_2_2^-ΔΔCt^) | H_2_O_2_  (log_2_2^-ΔΔCt^) | DFO  (log_2_2^-ΔΔCt^) | 17β-estradiol  (log_2_2^-ΔΔCt^) | H_2_O_2_  (log_2_2^-ΔΔCt^) | DFO  (log_2_2^-ΔΔCt^) |  |
| *HAT genes* | *^1^HAT1* | 0.87 | 0.93 | 0.43 | 0.88 | 1.74 | 1.90 |  |
|  | *^1^KAT5* | 1.36 | 1.81 | 2.80 | 1.27 | 1.29 | 1.83 |  |
|  | *^1^MYST1* | 0.46 | 0.79 | 1.15 | 0.21 | 0.42 | 1.10 |  |
|  | *EP300* | 0.38 | 0.87 | 0.71 | 0.41 | 0.89 | 0.91 |  |
|  | *NCOA1* | 0.20 | 0.42 | 0.88 | 0.25 | 0.52 | 0.73 |  |
|  | *KAT2A* | 0.86 | 1.37 | 1.97 | 0.27 | 0.13 | 0.69 |  |
|  | *MYST3* | 0.04 | 0.33 | 0.33 | 0.09 | 0.31 | 0.53 |  |
|  | *CREBBP* | 0.01 | 0.20 | 0.19 | -0.23 | 0.00 | 0.20 |  |
|  | *KAT2B* | 0.30 | -0.19 | 0.55 | 0.01 | -0.21 | 0.03 |  |
|  | *MYST2* | -0.02 | 0.13 | 0.31 | -0.11 | 0.19 | -0.08 |  |
|  | *ATF2* | -0.03 | -0.30 | 0.63 | 0.11 | -0.13 | -0.24 |  |
|  | *NCOA3* | -0.26 | 0.07 | 0.36 | -0.07 | -0.15 | -0.38 |  |
| *HDAC genes* | *^1^HDAC9* | 0.46 | 1.20 | 2.28 | 1.55 | 2.95 | 2.87 |  |
|  | *^1^SIRT1* | 0.75 | 1.36 | 1.86 | 1.07 | 2.00 | 2.37 |  |
|  | *SIRT4* | 0.39 | 0.59 | 0.10 | 0.42 | 0.96 | 1.11 |  |
|  | *SIRT5* | 0.19 | 0.40 | 0.29 | 0.27 | 0.29 | 0.80 |  |
|  | *HDAC4* | 0.01 | 0.40 | 1.12 | 0.17 | -0.48 | 0.71 |  |
|  | *HDAC11* | 0.69 | 0.70 | 1.35 | 0.16 | 0.06 | 0.48 |  |
|  | *SIRT2* | 0.04 | 0.60 | 0.66 | -0.12 | -0.34 | 0.38 |  |
|  | *HDAC2* | 0.30 | 0.51 | -0.43 | 0.06 | 0.58 | 0.28 |  |
|  | *SIRT7* | -0.01 | 0.34 | -0.25 | -0.39 | -0.49 | -0.25 |  |
|  | *SIRT3* | 0.06 | 0.21 | -0.56 | -0.40 | -0.87 | -0.54 |  |
|  | *HDAC8* | 0.21 | -0.23 | -0.36 | 0.29 | -0.37 | -0.55 |  |
|  | *HDAC3* | 0.07 | -0.21 | -0.46 | -0.24 | -0.34 | -0.64 |  |
|  | *HDAC1* | -0.11 | -0.16 | -0.52 | -0.34 | -0.53 | -0.67 |  |
|  | *HDAC5* | 0.04 | 0.90 | 1.49 | -0.12 | -1.06 | -0.77 |  |
|  | *^1^SIRT6* | -0.04 | 0.32 | -0.08 | -0.94 | -1.24 | -0.78 |  |
|  | *HDAC10* | -0.33 | 0.08 | -0.07 | -0.59 | -1.45 | -1.29 |  |
|  | *^1^HDAC7* | -0.12 | -0.41 | -0.39 | -0.70 | -1.26 | -1.52 |  |
|  | *^1^HDAC6* | -0.14 | -0.31 | -0.66 | -0.62 | -1.75 | -2.28 |  |
| *DNA methylation-associated genes* | | *ZBTB33* | 0.37 | 0.80 | 0.90 | 0.24 | 0.96 | 1.34 |
|  |  | *MBD4* | 0.18 | 0.55 | 0.30 | 0.22 | 0.63 | 0.98 |
|  |  | *^1^MBD1* | 0.92 | 1.49 | 0.67 | -0.06 | 0.26 | 0.85 |
|  |  | *MBD2* | 0.18 | 0.48 | 0.35 | 0.01 | 0.47 | 0.38 |
|  |  | *MECP2* | -0.12 | 0.00 | -0.12 | -0.41 | -0.43 | -0.13 |
|  |  | *DNMT1* | -0.01 | 0.40 | -0.03 | -0.29 | -0.16 | -0.16 |
|  |  | *MBD3* | 0.14 | 0.15 | 0.32 | -0.71 | -1.32 | -0.52 |
|  |  | *DNMT3B* | 0.05 | 0.30 | -0.07 | -0.24 | -0.21 | -0.83 |
|  |  | *^1^DNMT3A* | 0.19 | 0.28 | 0.58 | -0.54 | -1.77 | -1.88 |
| *OB-associated genes* | | *RANKL* | -0.11 | 0.25 | 0.27 | 0.53 | 2.03 | 0.43 |
|  |  | *OPG* | 0.18 | -0.01 | -0.49 | -0.23 | -0.45 | -0.77 |
|  |  | *LRP5* | -0.11 | 0.34 | 0.68 | -0.61 | -0.96 | -0.97 |
|  |  | *ALPL* | 0.06 | -0.05 | 0.25 | -0.14 | -0.39 | -1.93 |

**Supplementary Table S1.** Results of gene expression profiling of HOS cells

DFO treatment induced the most pronounced changes in the expression of analysed genes, followed by hydrogen peroxide and 17β-estradiol. There were also many more affected genes at the 72-hour mark as compared to the 24-hour time point. Data is presented as a binary logarithm of fold change of expression of each gene after the transfection of HOS cells with pCMV-ESR1 and exposure to the indicated treatment relative to the vehicle treated control. Only one biological replicate was used for the gene expression profiling purposes. Genes indicated with ^1^ were chosen for individual qPCR analyses. Preselected reference genes and *SOST* were excluded from the table.

|  | PMO (n=43) | OA (n=41) | controls (n=12) |
| --- | --- | --- | --- |
| Age (years) | 75.8 ± 6.3^1,2^ | 71.7 ± 7.1^1^ | 69.9 ± 11.2^2^ |
| Sex (women/men) | 35/8^2^ | 31/10^3^ | 2/10^2,3^ |
| BMI (kg/m^2^) | 25.0 ± 3.0^1^ | 28.8 ± 4.2^1,3^ | 25.2 ± 3.1^3^ |
| Hip BMD (g/cm^2^) | 0.709 ± 0.150^1^ | 0.883 ± 0.135^1^ | NA |
| Hip *t-score* | -2.260 ± 1.078^1^ | -0.872 ± 0.990^1^ | NA |
| Femoral neck BMD (g/cm^2^) | 0.604 ± 0.109^1^ | 0.767 ± 0.131^1^ | NA |
| Femoral neck *t-score* | -2.739 ± 0.952^1^ | -1.404 ± 1.135^1^ | NA |
| Lumbar spine BMD (g/cm^2^) | 0.843 ± 0.174^1^ | 0.977 ± 0.193^1^ | NA |
| Lumbar spine *t-score* | -1.949 ± 1.537^1^ | -0.758 ± 1.680^1^ | NA |

**Supplementary Table S2.** Anthropometric parameters of the patients included in the study

All values except for gender are presented as mean ± SD. ^1^ denotes p ≤ 0.05 in the case of PMO vs. OA; ^2^ denotes p ≤ 0.05 in the case of PMO vs. controls; ^3^ denotes p ≤ 0.05 in the case of OA vs. controls.

| Gene abbreviation | Gene name | Function |
| --- | --- | --- |
| *ALPL* | alkaline phosphatase, liver/bone/kidney | hydrolyzation of phosphoric esters and transphosphorylation reactions; involved in bone mineralization; marker of osteoblast activity |
| *AOX1* | aldehyde oxidase 1 | oxidation of a number of substrates and xenobiotics; involved in the regulation of reactive oxygen species homeostasis; marker of oxidative stress |
| *ATF2* | activating transcription factor 2 | transcriptional activation; acetylation of histones |
| *CREBBP* | CREB binding protein | transcriptional coactivation of many different transcription factors; acetylation of histone and non-histone proteins |
| *DNMT1* | DNA methyltransferase 1 | methylation of cytosine residues in DNA; responsible for maintaining methylation patterns following DNA replication |
| *DNMT3A* | DNA methyltransferase 3 alpha | methylation of cytosine residues in DNA; responsible for de novo methylation |
| *DNMT3B* | DNA methyltransferase 3 beta | methylation of cytosine residues in DNA; responsible for de novo methylation |
| *EP300* | E1A binding protein p300 | transcriptional coactivation; acetylation of histones |
| *ESR1* | estrogen receptor 1 (alpha) | nuclear steroid hormone receptor; transcriptional activation; involved in sexual development and reproductive function |
| *ESR2* | estrogen receptor 2 (beta) | nuclear steroid hormone receptor; transcriptional activation; involved in reproductive function |
| *GPER1* | G protein-coupled estrogen receptor 1 | estrogen-mediated rapid nongenomic intracellular signalling |
| *HAT1* | histone acetylase 1 | rapid acetylation of newly synthesized histones |
| *HDAC1* | histone deacetylase 1 | transcriptional repression; deacetylation of histone and non-histone proteins |
| *HDAC2* | histone deacetylase 2 | transcriptional repression; deacetylation of histone and non-histone proteins |
| *HDAC3* | histone deacetylase 3 | transcriptional repression; deacetylation of histone and non-histone proteins |
| *HDAC4* | histone deacetylase 4 | transcriptional repression; deacetylation of histone and non-histone proteins |
| *HDAC5* | histone deacetylase 5 | transcriptional repression; deacetylation of histone and non-histone proteins |
| *HDAC6* | histone deacetylase 6 | transcriptional repression; deacetylation of histone and non-histone proteins; involved in degradation of misfolded proteins |
| *HDAC7* | histone deacetylase 7 | transcriptional repression; deacetylation of histone and non-histone proteins |
| *HDAC8* | histone deacetylase 8 | transcriptional repression; deacetylation of histone and non-histone proteins |
| *HDAC9* | histone deacetylase 9 | transcriptional repression; deacetylation of histone and non-histone proteins |
| *HDAC10* | histone deacetylase 10 | transcriptional repression; deacetylation of histone and non-histone proteins |
| *HDAC11* | histone deacetylase 11 | transcriptional repression; deacetylation of histone and non-histone proteins |
| *HIF1α* | hypoxia inducible factor 1 subunit alpha | primary transcriptional activator induced by hypoxia; orchestrates metabolic adaptation to hypoxia; involved in energy metabolism, angiogenesis, apoptosis |
| *KAT2A* | lysine acetyltransferase 2A | transcriptional activation; acetylation of histone and non-histone proteins |
| *KAT2B* | lysine acetyltransferase 2B | transcriptional activation; acetylation of histone and non-histone proteins |
| *KAT5* | lysine acetyltransferase 5 | transcriptional activation; acetylation of histone and non-histone proteins; involved in DNA repair and apoptosis |
| *KMT2D* | lysine-specific methyltransferase 2D | transcriptional activation; methylation of histones |
| *LRP5* | LDL receptor related protein 5 | transmembrane low-density lipoprotein receptor; coreceptor in the WNT/β-catenin signalling pathway; involved in the regulation of osteoblast function and bone formation |
| *MBD1* | methyl-CpG binding domain protein 1 | transcriptional repression; can bind specifically to methylated DNA; recruits histone methyltransferases |
| *MBD2* | methyl-CpG binding domain protein 2 | transcriptional repression; can bind specifically to methylated DNA; recruits histone deacetylases and DNA methyltransferases |
| *MBD3* | methyl-CpG binding domain protein 3 | transcriptional repression; not capable of binding to methylated DNA by itself; recruits histone deacetylases and DNA methyltransferases |
| *MBD4* | methyl-CpG binding domain protein 4 | can bind specifically to methylated DNA; mismatch-specific DNA glycosylase activity; involved in DNA repair |
| *MECP2* | methyl-CpG binding protein 2 | transcriptional repression; can bind specifically to methylated DNA |
| *MEF2* | myocyte enhancer factor 2 | transcriptional activation; DNA-binding transcription factor |
| *MYST1* | lysine acetyltransferase 8 (KAT8) | transcriptional activation;  acetylation of histone and non-histone proteins |
| *MYST2* | lysine acetyltransferase 7 (KAT7) | transcriptional activation;  acetylation of histones |
| *MYST3* | lysine acetyltransferase 6A (KAT6A) | transcriptional activation;  acetylation of histone and non-histone proteins |
| *NCOA1* | nuclear receptor coactivator 1 | transcriptional coactivator for steroid and nuclear hormone receptors; acetylation of histones |
| *NCOA3* | nuclear receptor coactivator 3 | transcriptional coactivator for nuclear hormone receptors; acetylation of histones |
| *OPG* | osteoprotegerin | osteoblast-secreted decoy receptor for RANKL; negative regulator of osteoclastogenesis and bone resorption |
| *RANK* | receptor activator of NF-κB | receptor for RANKL; essential for RANKL-mediated osteoclastogenesis and bone resorption |
| *RANKL* | receptor activator of NF-κB ligand | ligand for OPG and RANK; key factor for osteoclast differentiation and activation and bone resorption |
| *SIRT1* | sirtuin 1 | transcriptional repression or activation depending on the target protein; deacetylation of histone and non-histone proteins; involved in the regulation of intracellular energy status |
| *SIRT2* | sirtuin 2 | deacetylation of histone and non-histone proteins; involved in cell cycle and genomic stability control; involved in VEGFA and HIF1A regulation |
| *SIRT3* | sirtuin 3 | deacetylation of proteins; involved in the regulation of cellular energy metabolism |
| *SIRT4* | sirtuin 4 | lipoamidase and ADP-ribosyl transferase activity; deacetylation of proteins |
| *SIRT5* | sirtuin 5 | demalonylation, desuccinylation deglutarylation and deacetylation of proteins |
| *SIRT6* | sirtuin 6 | transcriptional repression; ADP-ribosyl transferase activity; deacetylation of histones; involved in the maintenance of genomic stability and glucose homeostasis |
| *SIRT7* | sirtuin 7 | transcriptional repression; selective deacetylation of histone mark H3K18Ac |
| *SOST* | sclerostin | inhibition of WNT/β-catenin signalling pathway; negative regulation of bone formation |
| *VEGFA* | vascular endothelial growth factor A | growth factor involved in angiogenesis; direct HIF1A downstream target gene |
| *ZBTB33* | zinc finger and BTB domain containing 33 | transcriptional repression or activation depending on the context; can bind specifically to methylated and non-methylated DNA |

**Supplementary Table S3.** A list of genes analysed in the study

| Gene | Forward primer sequence (5'-3') | Reverse primer sequence (5'-3') | Reference |
| --- | --- | --- | --- |
| *KAT5* | GGGAGGTGGGTAGAGCCC | GCCAGGGGCCACTCAT | self-designed |
| *HAT1* | CGGAAATGGCGGGATTTGGT | AGTTTCTTCTCCACTGCACTCT | self-designed |
| *MYST1* | CGGATAGCACCTGGCATTCT | CCGGTTAAAGCCCACGTAGT | self-designed |
| *HDAC6* | GATCTGGCGGAGTGGAAGAACC | TTCTTCGCTGCCTGGTTGTG | self-designed |
| *HDAC7* | ACCTGGCTGCTTTCAGGATA | TCCAGCAGACACCAGGACTA | self-designed |
| *HDAC9* | TGTAGCTGGTGGAGTTCCCT | CTCTGAGGCAAAGGTGCAGA | self-designed |
| *SIRT1* | TCTAACTGGAGCTGGGGTGT | TCTACAGCAAGGCGAGCATAA | self-designed |
| *SIRT6* | TTCCCCAGGGACAAACTGGC | GCGTCTTACACTTGGCACATTC | self-designed |
| *DNMT3A* | GAAGGACTTGGGCATTCAG | CCGACGTACATGATCTTCCC | self-designed |
| *MBD1* | ACCATGAATCCGCTCCCAAG | TCCATGGTCTTCAGCTTTGC | self-designed |
| *AOX1* | AAATAGACTGCCTGACGGGG | TGCACCTTCAATCTGGCCTA | self-designed |
| *HIF1α* | CGAAGTAGTGCTGACCCTGC | GGACTATTAGGCTCAGGTGAACT | self-designed |
| *VEGFA* | AGCCTTGCCTTGCTGCTCTACC | GTGATGATTCTGCCCTCCTCCTTC | [^2^](#_ENREF_2) |
| *AXIN2* | AGTGTGAGGTCCACGGAAAC | TGGCTGGTGCAAAGACATAG | [^3^](#_ENREF_3) |
| *OPG* | gaagggcgctaccttgagat | gcaaactgtatttcgctctgg | self-designed |
| *RANKL* | tgattcatgtaggagaattaaacagg | gatgtgctgtgatccaacga | self-designed |
| *ESR1* | GAATCTGCCAAGGAGACTCGC | ACTGGTTGGTGGCTGGACAC | [^4^](#_ENREF_4) |
| *ESR2* | TGTCTGCAGCGATTACGCA | GCGCCGGTTTTTATCGATT | [^4^](#_ENREF_4) |
| GPER1 | TTCTGAGGAAGACCCACCCC | TGAATTTTCACTCGCCGCTTC | self-designed |
| *MYST4* | CGTTTGAAGTTCCAATTCTGGTCT | AGGAAAGACTGCATTCGGGT | self-designed |
| *RPLP0* | TCTACAACCCTGAAGTGCTTGAT | CAATCTGCAGACAGACACTGG | [^5^](#_ENREF_5) |
| *GAPDH* | TGCACCACCAACTGCTTAGC | TGGCATGGACTGTGGTCATG | [^6^](#_ENREF_6) |

**Supplementary Table S4**. Primer pair sequences used for individual qPCR analyses

| Primer name | Forward primer sequence (5'-3') | Reverse primer sequence (5'-3') |
| --- | --- | --- |
| Meth-OPG | TTCGGATTTTGGTTGGATC | GCAAACTCTAAAATTTCCCG |
| UNmeth-OPG | GTTTGGATTTTGGTTGGATT | CACAAAACTCTAAAATTTCCCA |
| Meth-RANKL | AGGAGATGGGCGTTTTTAGTC | CCGCTCCATATTCGTAACC |
| UNmeth-RANKL | GGAAGGAGATGGGTGTTTTTAGTT | CCCACTCCATATTCATAACCCT |
| GAPDH_TpG | GGTTTTTGGTATTGTAGGTTTT | CCAATTACAACATAACAACCA |
| OPG 1 | GGAAAGGGGCACATCCAGAA | CAGGCAGTGAGTGGCTGAAT |
| OPG 2 | TCCCCAGTGACTTTTTGGGC | ACGCCCGCTATTTCACAGAA |
| OPG 3 | TCAGGTGGATAGCACCTCACA | AGGCCTTGAATGTCAGACACA |
| OPG 4 | TTGTCTGAGCCCTTTTCGGG | ACGGGCTTTAGGGCCAATC |
| RANKL 1 | GATAAGCAGTGGGCGAGGTT | CTATGCCACCAGAGAGGCAG |
| RANKL 2 | GGGAAACAACACTGGCCTCT | TAGATGCCCAAGTGAAGGCG |
| RANKL 3 | ACAAAGAGGGGCGTATCCTT | CTAGGGCCACTCAGGAGAGAT |

**Supplementary Table S5.** Primer pair sequences used for qMSP and ChIP analyses

**References**

1 Delgado-Calle, J. *et al.* Role of DNA methylation in the regulation of the RANKL-OPG system in human bone. *Epigenetics*. **7**, 83-91 (2012).

2 el Filali, M. *et al.* Regulation of VEGF-A in uveal melanoma. *Investigative ophthalmology & visual science*. **51**, 2329-2337 (2010).

3 Obrador-Hevia, A. *et al.* Oncogenic KRAS is not necessary for Wnt signalling activation in APC-associated FAP adenomas. *The Journal of pathology*. **221**, 57-67 (2010).

4 Stossi, F. *et al.* Transcriptional profiling of estrogen-regulated gene expression via estrogen receptor (ER) alpha or ERbeta in human osteosarcoma cells: distinct and common target genes for these receptors. *Endocrinology*. **145**, 3473-3486 (2004).

5 Dragojevic, J. *et al.* Triglyceride metabolism in bone tissue is associated with osteoblast and osteoclast differentiation: a gene expression study. *J Bone Miner Metab*. **31**, 512-519 (2013).

6 Vandesompele, J. *et al.* Accurate normalization of real-time quantitative RT-PCR data by geometric averaging of multiple internal control genes. *Genome biology*. **3**, RESEARCH0034 (2002).

**
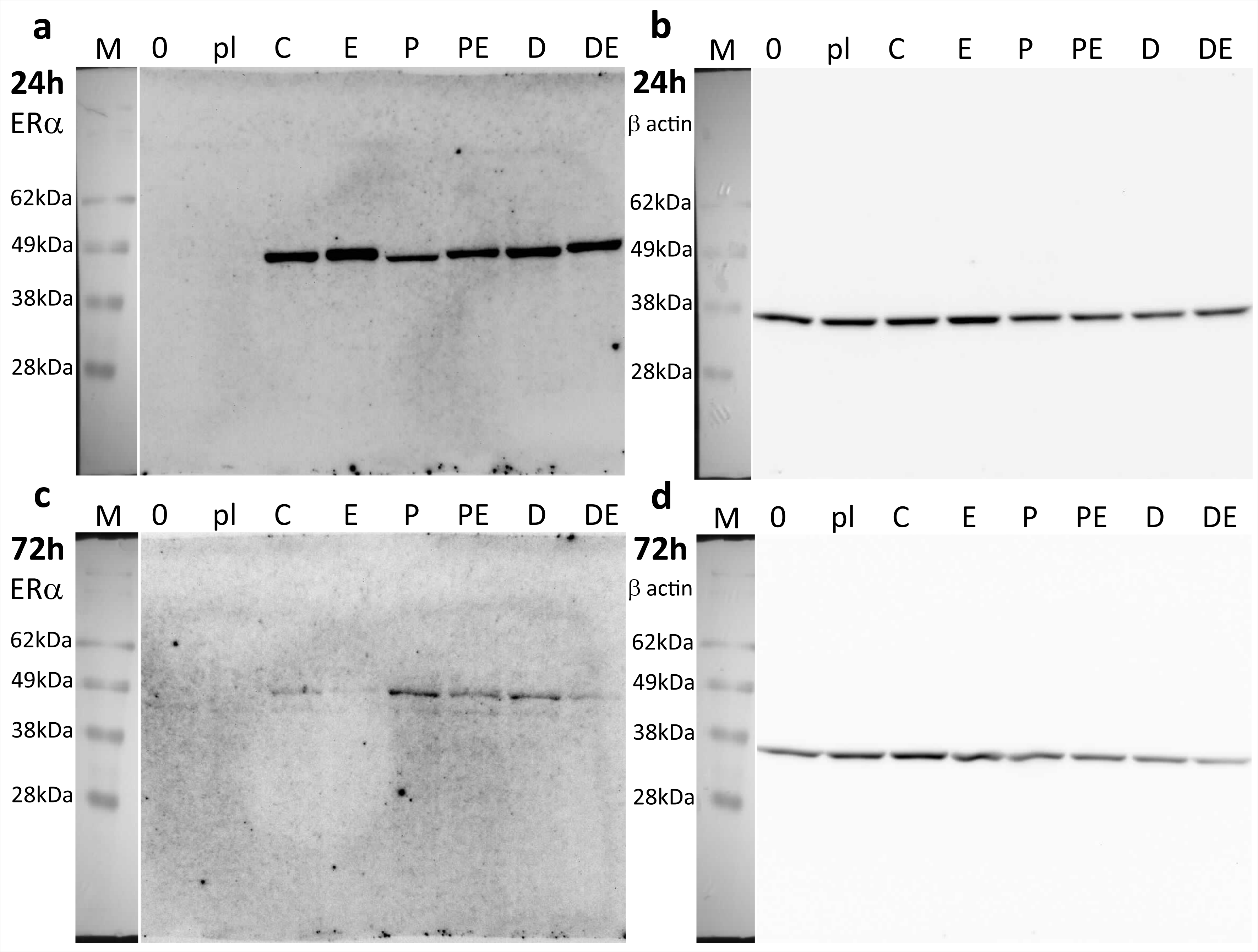
**

**Supplementary Figure 1. The presence of ERα protein in HOS cells after transfection.** Western blot analysis of proteins isolated from HOS cells transfected with pCMV-ESR1 and treated with vehicle control – C; 10 nM 17β-estradiol – E; 500 μM hydrogen peroxide – P; 500 μM hydrogen peroxide and 10 nM 17β-estradiol – PE; 20 µM DFO – D; 20 µM DFO and 10 nM 17β-estradiol – DE. No plasmid vehicle control – 0; as well as cells transfected with an empty vector and treated with vehicle control – pl; were also performed. Each of the blot images is assembled from a protein ladder – M imaged under visual light while in the rest of the lanes chemiluminescent signal was captured. (**a**, **b**) indicates a 24- and (**c**, **d**) a 72-hour treatment period. ERα (**a**, **c**) was compared to β actin (**b**, **d**) loading control. Transfection control was performed for each of the three biological replicates; however only representative results are shown.

**
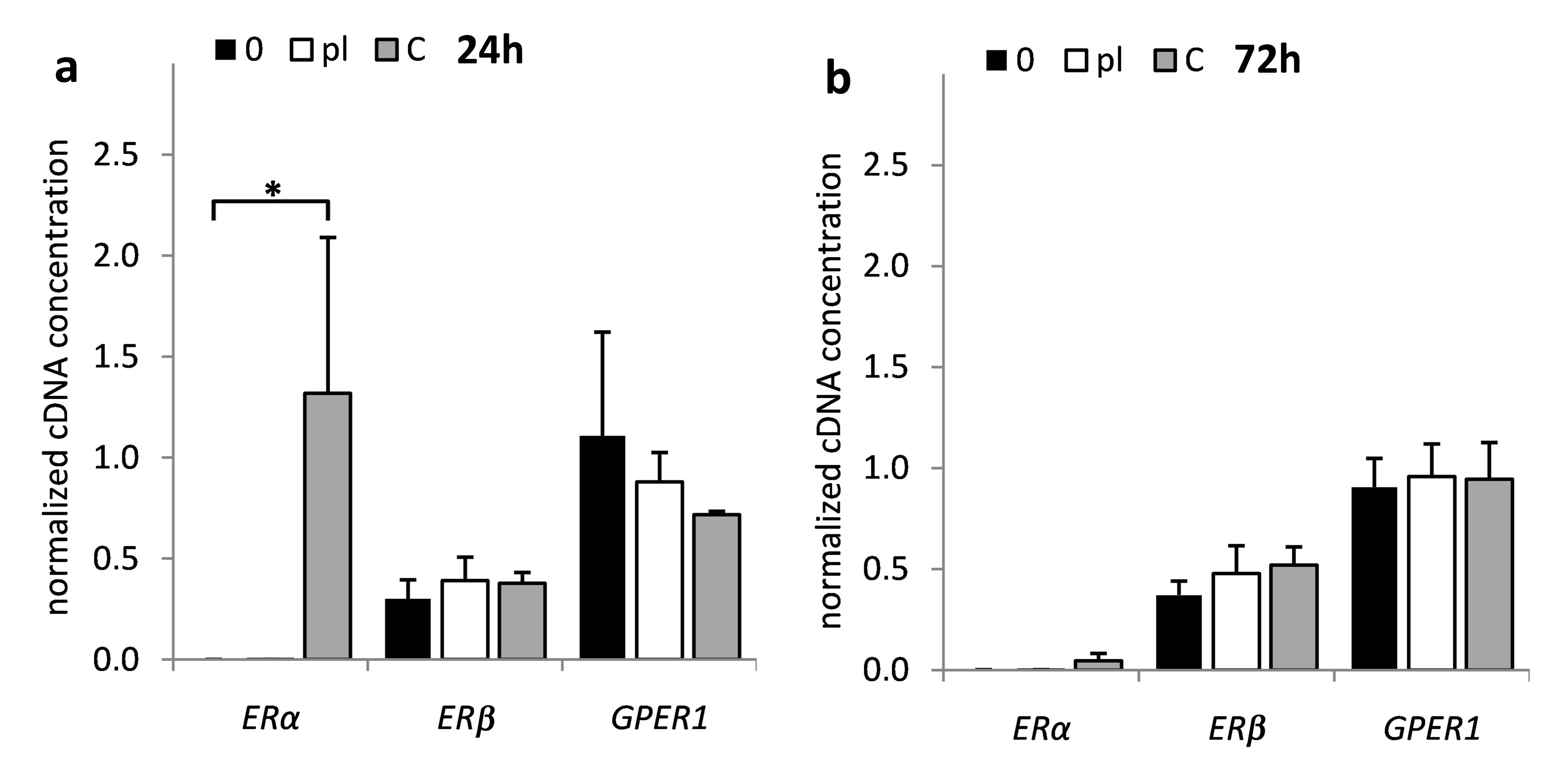
**

**Supplementary Figure 2. *ERα*, *ERβ* and *GPER1* gene expression in HOS cells before and after transfection with pCMV-ESR1.** HOS cells were transfected with pCMV-ESR1 – C, empty vector – pl, or used as a no plasmid control – 0 and treated with vehicle control for other experimental conditions for (**a**) 24 and (**b**) 72 hours. Values are presented as mean ± SD of normalized cDNA concentrations (n = 3). * denotes p ≤ 0.05 as compared to the empty vector and no plasmid control.

**
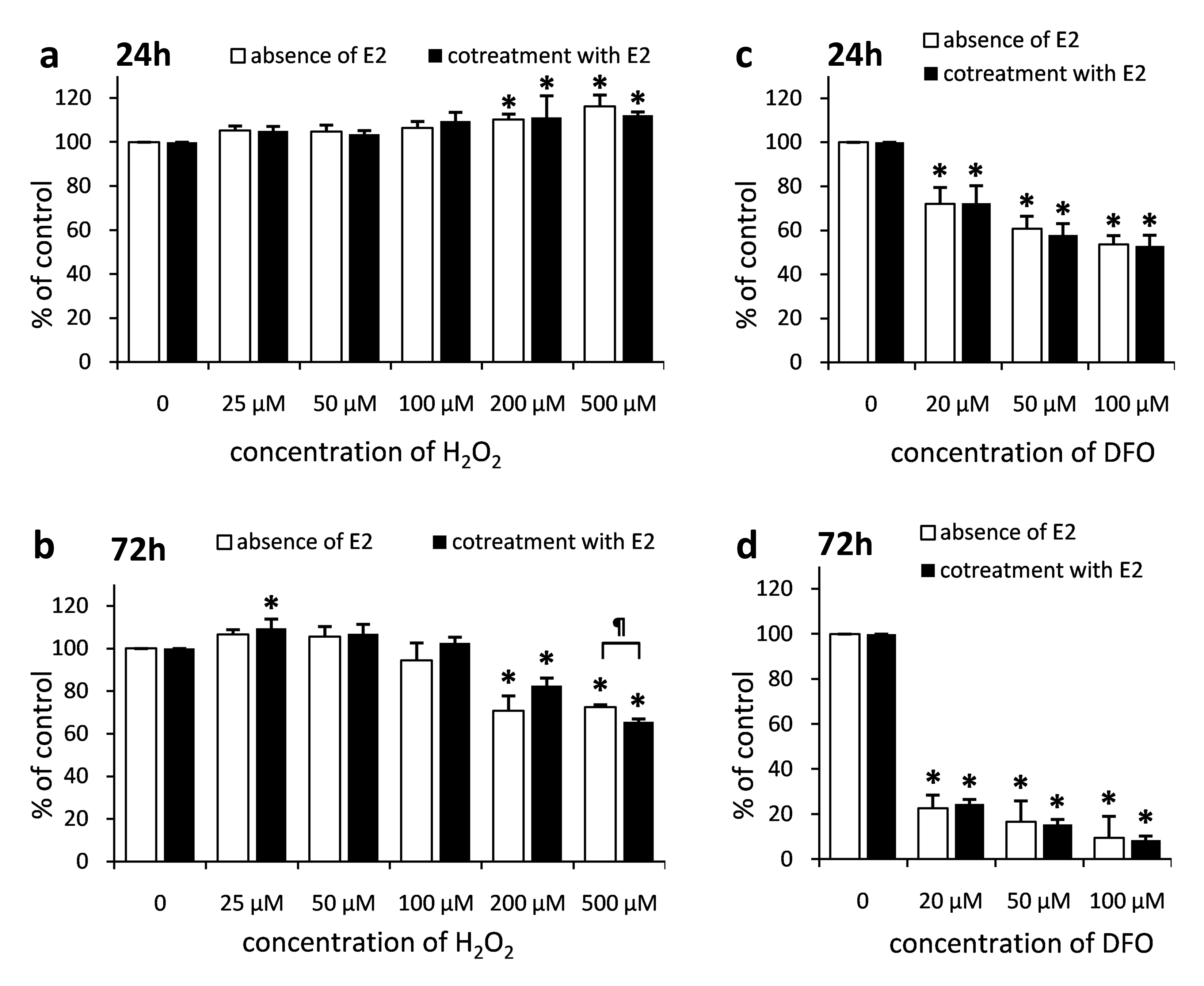
**

**Supplementary Figure 3. The influence of hydrogen peroxide and DFO on the viability of HOS cells.** HOS cell were transfected with pCMV-ESR1 and treated with increasing concentrations of hydrogen peroxide for 24 (**a**) and 72 (**b**) hours or DFO for 24 (**C**) and 72 (**D**) hours or vehicle control in the presence or absence of 10 nM 17β-estradiol. Values are presented as mean ± SD of the viability of treated cells relative to vehicle control cells (n = 3). * denotes p ≤ 0.05 as compared to the vehicle control, while ^¶^ denotes p ≤ 0.05 as compared to the 17β-estradiol treated parallel. E2, 17β-estradiol.

**
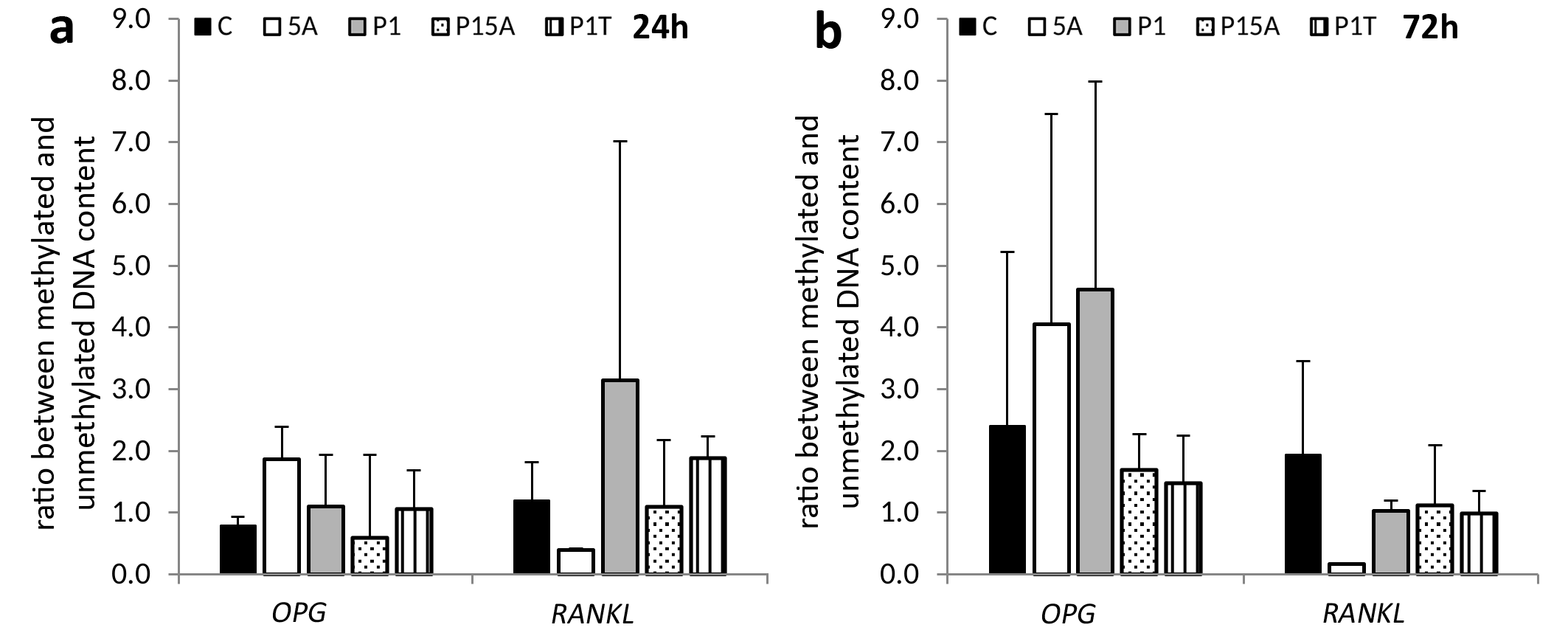
**

**Supplementary Figure 4: The influence of H_2_O_2_ (100 μM)**, **5-azacytidine and tempol on the ratio between methylated and unmethylated DNA at *OPG* and *RANKL* promoters in HOS cells.** HOS cells were treated for 24 (**a**) or 72 (**b**) hours with vehicle control – C, H2O2 alone – P1 or in the presence of 2.5 μM 5-azacytidine – P15A or 200 μM tempol – P1T. Values are presented as mean ± SD of the ratio between methylated and unmethylated DNA content (n = 3).

**
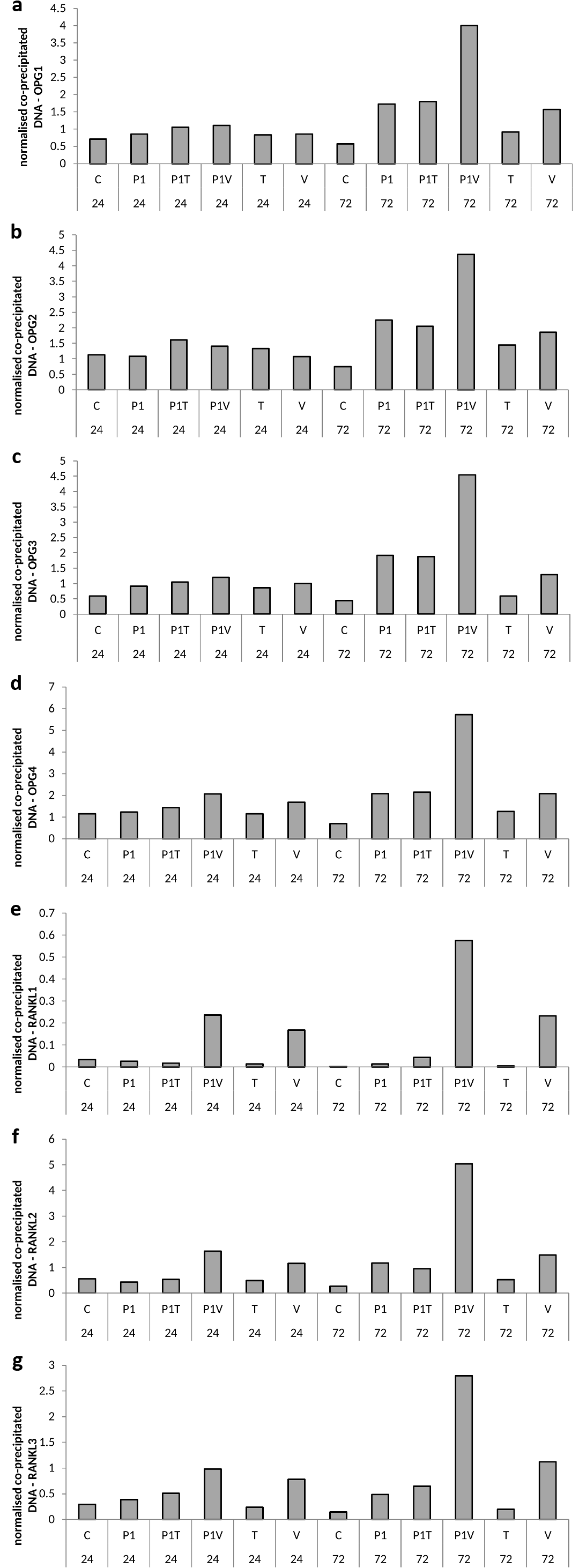
**

**Supplementary Figure 5: The influence of H_2_O_2_ (100 μM), vorinostat and tempol on the levels of histone H3 acetylation at four *OPG* and three *RANKL* sites in HOS cells.** HOS cells were treated for 24 or 72 hours with vehicle control – C, 0.625 μM vorinostat – V, 100 μM H2O2 alone – P1 or in the presence of 0.625 μM vorinostat – P1V or 200 μM tempol – P1T. Values from a single experiment are shown as normalized co-precipitated DNA concentrations (n = 1).
